# Chemical carcinogen safety testing: OECD expert group international consensus on the development of an integrated approach for the testing and assessment of chemical non-genotoxic carcinogens

**DOI:** 10.1007/s00204-020-02784-5

**Published:** 2020-06-27

**Authors:** Miriam N. Jacobs, Annamaria Colacci, Raffaella Corvi, Monica Vaccari, M. Cecilia Aguila, Marco Corvaro, Nathalie Delrue, Daniel Desaulniers, Norman Ertych, Abigail Jacobs, Mirjam Luijten, Federica Madia, Akiyoshi Nishikawa, Kumiko Ogawa, Kiyomi Ohmori, Martin Paparella, Anoop Kumar Sharma, Paule Vasseur

**Affiliations:** 1 Centre for Radiation, Chemical and Environmental Hazards (CRCE), Public Health England, Chilton, UK; 2 Center for Environment, Prevention and Health, Regional Agency for Prevention, Environment and Energy Emilia Romagna Region (Arpae), Bologna, Italy; 3 European Commission Joint Research Centre (EC JRC), Ispra, Italy; 4 US Food and Drug Administration (FDA), Silver Spring, MD USA; 5 Corteva Agriscience, Abingdon, UK; 6 Organisation for Economic Cooperation and Development (OECD), Paris, France; 7 Health Canada, Ottawa, Canada; 8 German Centre for the Protection of Laboratory Animals (Bf3R), German Federal Institute for Risk Assessment, Diedersdorfer Weg 1, 12277 Berlin, Germany; 9 National Institute for Public Health and the Environment (RIVM), Bilthoven, Netherlands; 10 National Institute of Health Sciences, Kawasaki, Japan; 11 Kanagawa Prefectural Institute of Public Health, Chigasaki, Japan; 12 Division of Medical Biochemistry, Biocenter, Medical University of Innsbruck, Innsbruck, Austria; 13 Technical University of Denmark, Lyngby, Denmark; 14 CNRS University de Lorraine, Lorraine, France

**Keywords:** Non-genotoxic carcinogenicity, Integrated approaches to testing and assessment, IATA, Cancer hallmarks, Hazard assessment, Cancer prevention, Cancer model, Cancer microenvironment

## Abstract

While regulatory requirements for carcinogenicity testing of chemicals vary according to product sector and regulatory jurisdiction, the standard approach starts with a battery of genotoxicity tests (which include mutagenicity assays). If any of the in vivo genotoxicity tests are positive, a lifetime rodent cancer bioassay may be requested, but under most chemical regulations (except plant protection, biocides, pharmaceuticals), this is rare. The decision to conduct further testing based on genotoxicity test outcomes creates a regulatory gap for the identification of non-genotoxic carcinogens (NGTxC). With the objective of addressing this gap, in 2016, the Organization of Economic Cooperation and Development (OECD) established an expert group to develop an integrated approach to the testing and assessment (IATA) of NGTxC. Through that work, a definition of NGTxC in a regulatory context was agreed. Using the adverse outcome pathway (AOP) concept, various cancer models were developed, and overarching mechanisms and modes of action were identified. After further refining and structuring with respect to the common hallmarks of cancer and knowing that NGTxC act through a large variety of specific mechanisms, with cell proliferation commonly being a unifying element, it became evident that a panel of tests covering multiple biological traits will be needed to populate the IATA. Consequently, in addition to literature and database investigation, the OECD opened a call for relevant assays in 2018 to receive suggestions. Here, we report on the definition of NGTxC, on the development of the overarching NGTxC IATA, and on the development of ranking parameters to evaluate the assays. Ultimately the intent is to select the best scoring assays for integration in an NGTxC IATA to better identify carcinogens and reduce public health hazards.

**Electronic supplementary material:**

The online version of this article (10.1007/s00204-020-02784-5) contains supplementary material, which is available to authorized users.

## Introduction

Chemicals (both anthropogenic and natural) that can cause cancer can be classed into two types: genotoxic and non-genotoxic (BOX 1 “Terminology”). Some carcinogens are inherently genotoxic but also contribute to carcinogenesis through non-genotoxic mechanisms. In contrast, non-genotoxic carcinogens are not inherently genotoxic in short-term assays. The purpose of chemical regulation is to safely control chemical hazards to ensure the protection of human health and the environment, whilst also supporting innovation.

## Box 1. Terminology

### Definitions of genotoxic carcinogenicity and non-genotoxic carcinogenicity, mode of action, and IATAs for regulatory purposes.

The OECD expert group consensus agreed definition of **NGTxC**/indirect carcinogenesis was adapted from (Adler et al. 2011), and is as follows:

*The induction of cancer involves the accumulation of genomic alterations, which can be induced directly or indirectly. Carcinogens have conventionally been divided into two categories according to their presumed mode of action, genotoxic*^1^*carcinogens and non-genotoxic carcinogens. A genotoxic carcinogen has the potential to induce cancer by interacting directly with DNA and/or the cellular apparatus involved in the preservation of the integrity of the genome. A non-genotoxic carcinogen has the potential to induce cancer without interacting directly with either DNA or the cellular apparatus involved in the preservation of the integrity of the genome.*

**Mode of action**,* is a biologically plausible series of key events leading to an effect* (Sonich-Mullin et al. 2001). *Originally, the context of mode of action was in relation to late-stage key cellular, biochemical and tissue events. A key event is an empirically observable step or its marker, which is a necessary element of the mode of action critical to the outcome (i.e. necessary, but not necessarily sufficient in its own right); key events are measurable and reproducible. Early key events are usually related to chemical characteristics such as those of structure and/or physicochemical properties that enable interaction of the substance with biological targets. Later key events are less chemical specific but are a likely expected consequence of the progression of the earlier key events (e.g. regenerative proliferation resulting from cytotoxicity* (Meek et al. 2014). *Mechanism of action provides a more detailed level of understanding compared to mode of action.*

**An adverse outcome pathway (AOP)** *describes existing knowledge on the toxicity mechanisms at different levels of biological organization that lead to an adverse human and/or environmental health effect* (Ankley et al. 2010; OECD 2017a).

**An Integrated Approach to Testing and Assessment (IATA)** *is a structured approach used for hazard identification (potential), hazard characterization (potency) and/or safety assessment (potential/potency and exposure) of a chemical or group of chemicals, which strategically integrates and weights all relevant data to inform regulatory decision regarding potential hazard and/or risk and/or the need for further targeted testing and therefore optimizing and potentially reducing the number of tests that need to be conducted* (OECD 2016a).

Understanding the carcinogenic potential of chemicals is a critical aspect of regulatory assessment for human health risks. It is now well recognized by the scientific and regulatory community that the conventional approach to carcinogenicity testing, particularly the use of the rodent cancer bioassay (OECD 2018a, b) has many limitations in terms of reliability and relevance. It is not considered sufficiently fit for the purpose of human health hazard assessment (that is, assessment of hazardous properties, eventually including potencies and limit values) (Alden et al. 2011; Boobis et al.  2009, 2016; Gottmann et al. 2001; Paparella et al. 2016; Thayer and Foster 2007; UK Committee on Carcinogenicity of Chemicals in Food 2019), and critical Mode of Action (MoA) information providing insights into human relevance may be missing (Meek et al. 2014; WHO 2007). The rodent cancer bioassay is routely used for pharmaceutical, plant protection product and biocide safety evaluation. However, this information is not routinely available for environmental and industrial chemicals (Jacobs et al. 2016; Madia et al. 2019; Madia 2016; Woutersen et al. 2018). The utility of the rodent cancer bioassay has also been challenged in the broader context of risk assessment and alternatives suggested (Cohen 2004, 2010a, b, 2018; Goodman 2018; Marone et al. 2014).Whilst there are good batteries of genotoxicity and mutagenicity test methods available as OECD Test Guidelines (TGs) that have been used successfully for many years, there are no (in vitro) TGs that specifically address human-relevant non-genotoxic carcinogenicity. In recent years, the data on cancer incidence acknowledge cancer to be a serious health policy issue (Luijten et al. 2016; Madia et al. 2019). It is clear that there is an urgent need to deepen our understanding of the chemical contribution to cancer (both chemicals entering the market and the environment), in order to better protect public health, with more appropriate carcinogenicity testing methods, especially to address the multiple non-genotoxic mechanisms.

The global development and harmonization of methods for the testing of chemicals, such as OECD TGs 451, 453 (OECD 2018a, b), are conducted under the auspices of the OECD Test Guideline Programme (TGP). This programme specifically develops hazard assessment tools in the form of test methods, for the international regulatory community, to assess the hazardous properties and eventually related potencies and limit values of a test chemical, to better protect both the environment and human health, whilst also supporting innovation and green chemistry. The agreed harmonized validated methods and frameworks for assessing the safety of chemicals, enables the Mutual Acceptance of Data (MAD), thereby avoiding duplicative testing across all OECD member countries, greatly reducing both animal use and costs to industry. Test methods undergo validation and peer review with the intention to ultimately be consensually adopted by OECD member countries as TGs that fall under the MAD agreement. Chemical risk assessment varies according to sector and regulatory jurisdiction and is not part of the hazard tool development process, within the OECD TGP. Furthermore, the TGP does not include population exposure assessment (that changes throughout the life cycle), therefore the focus of the work reported herein does not include this.

Validated in vitro alternatives to the rodent cancer assay have been proposed. However in 2014, the validated in vitro cell transformation assays (CTAs) (Corvi et al. 2012) failed in Test Guideline adoption instead being adopted as guidance documents (OECD 2015, 2017b ). The 2014 meeting of the OECD Working Group of National Coordinators of the OECD Test Guidelines Programme (the intergovernmental representation group that has the mandate to oversee the TGP) considered that it was not sufficiently well understood and recognized that no single test can currently demonstrate and predict NGTxC (OECD 2015).

Instead, a battery of appropriate tests was needed, to address the limitations of the rodent cancer bioassay, together with the general lack of validated test methods and regulatory approaches available to assess non-genotoxic carcinogenicity (NGTxC). To examine how to address what these assays should determine, and how they should be integrated, an OECD steering group worked on the challenging task of building consensus and a vision for the concept of an NGTxC IATAthat could realistically accommodate different theories and approaches to cancer hazard assessment. This thought-starter was published in 2016 (Jacobs et al. 2016), including a table of major mechanisms of non-genotoxic carcinogenicity, and suggested data organization for IATA development is adapted here in the supplementary information Table 1.

Here, we present the consensus statement from the OECD NGTxC IATA expert group, regarding the process undertaken to derive the structure of the IATA agreed, which will next be used as a transparent basis to distil, evaluate and organize test methods and in vitro assays. In turn, this will facilitate future test guideline development for addressing the key event gaps in cancer hazard assessment.

## Methods: steps taken to develop the overarching IATA

In describing the methodological steps taken to achieve the development of the overarching NGTxC IATA, it is first necessary to explain the methodology required to establish this programme of work and the formation of the OECD expert group. Box 2 “Process for establishment of workplan projects and the supporting NGTxC IATA expert groups to the OECD Test Guideline Programme” summarizes the OECD process for the establishment of OECD workplan projects.

### Background context and methodology for the formation of the OECD NGTxC IATA expert group

In 2014, the OECD Working Group of National Coordinators of the Test Guideline Programme meeting (WNT) acknowledged that a battery of tests would be needed to assess NGTxC, and during this meeting, an OECD steering group was specifically formed to develop a provisional conceptual basis for how this could be done. The steering group membership represented the different and sometimes contrasting views of the WNT governmental representatives and the respective country experts, and included the European Chemicals Agency, the Netherlands, Italy, Austria, France and the UK (chair). The output of the regular structured discussions, held by teleconference and email, was the peer-reviewed publication of a thought-starter (Jacobs et al. 2016) that considered how to ensure coverage of the complexity of the carcinogenicity processes to meet regulatory needs and that could serve to facilitate the initiation of the work at the OECD. Establishing this base level was a challenging task, taking almost two years to build, as the views regarding the process of non-genotoxic carcinogenic disease initiation and progression, and how non-animal-based test methods can address these, are diverse, both within the WNT regulatory sphere and perhaps even more so in the wider cancer biology scientific community. The paper and project proposals were discussed with the WNT and accepted in 2016, as a basis to develop a consensus way forward, and an OECD expert group could then be established.

Whilst IATAs have been developed in recent years for less complex endpoints such as eye irritation, and skin sensitization, this was the first time that an IATA proposal for such a complex endpoint was accepted into the OECD TGP workplan.

## Box 2. Process for establishment of workplan projects and the supporting NGTxC IATA expert groups to the OECD Test Guideline Programme

The process for establishing an expert group at the OECD first requires that following two consultation reviews, a project proposal and work plan led by one or more OECD member countries is unanimously accepted by the OECD Working group of National Coordinators to the Test Guideline Programme (WNT). The National Coordinators coordinate on behalf of their respective national Governments. The WNT has the mandate to develop test guidelines that are applicable under the MAD principle. Several months before each face-to-face expert meeting, the OECD secretariat requested that the National Coordinators from each OECD member country nominate specific experts to join the expert group to address the work. In addition, the expert group invitation is extended to non-governmental OECD affiliated groups, principally the Business and Industry Advisory Council (BIAC) to the OECD TGP, and the International Council on Animal Protection in OECD programmes (ICAPO).

By working in this way, it is intended to involve and negotiate between relevant stakeholders, fostering mutual understanding and ensuring that bridges are built between regulators, academic and industry scientists, to target and accelerate the work consensually, such that all the stakeholders have ownership of the process and agree with the final recommendations. Provided there is consensus, and this is well communicated to the respective National Coordinators, it becomes possible to move ahead and approve the output at the OECD intra-governmental WNT level. At the WNT level and above, consensus needs to be achieved for OECD member countries.

For further information, see https://www.oecd.org/chemicalsafety/testing/oecd-guidelines-testing-chemicals-related-documents.htm

The initial activities of the expert group in the first two face to-face meetings (of 2-days duration each) and related teleconferences, held during 2016 and 2017 at the OECD headquarters in Paris, France, were to review and build upon the thought-starter by expanding upon a preliminary NGTxC assays database, to develop preliminary definitions, to examine uncertainties around the standard animal reference testing and assessment approaches and to review the global regulatory status of carcinogenicity testing and particularly NGTxC testing in OECD member countries. For the latter, this confirmed the lack of requirements for NGTxC mechanisms and MoAs and underlined the need for a globally harmonized IATA.

In order to achieve regulatory confidence in new approaches to carcinogenicity testing, an in-depth uncertainty characterization of standard animal reference testing and assessment approaches was conducted and further developed (Paparella et al. 2016). This is expected to provide an objective benchmark to facilitate the acceptance of the IATA being developed and test methods within. The intention here is not to aim for same level of information as the rodent cancer bioassay, but to improve upon it, by generating high-quality robust and predictive mechanistic and modality data for the endpoint/MoA at stake that could be utilized particularly by regulators to make legally binding regulatory decisions, ultimately within an integrated approach leading to a greater level of human health and environmental protection. Regulators have expressed a need for structured approaches that appropriately incorporate AOPs and preferably in vitro assays to investigate key events thereof, to assist with their risk assessments (Wittwehr et al. 2020).

### Background context and methodology for the development of a definition of NGTxC for regulatory purposes

Before further developing the work, it was necessary to come to a common place of understanding with respect to the definition of NGTxC. As part of the outcome of the third meeting held at the OECD headquarters in June 2018, the working definition of NGTxC agreed at the March 2017 meeting was revisited, and finally, a consensually agreed definition specifically to encompass the OECD member country regulatory needs was derived (BOX 1 “Terminology”).

Mechanistic insights as to how NGTxC induce accumulation of genomic alterations include, for example where sustained cell proliferative activity may cause genomic changes by chance, and also the possibility that oxidative DNA damage associated with inflammation or cytotoxicity may occur. For instance, whilst the MIE may not be a genotoxic event, it is recognized that secondary genotoxic events may occur following inflammation, and thus, according to the definition provided, these would be termed as NGTxC. The path taken forward was to develop an overarching IATA, having agreed the mode of action and hallmark assay blocks (total 13) necessary to populate the IATA (Jacobs et al. 2016). This involves striving for the coverage and addressing the interplay of all the hallmarks of cancer (Colotta et al. 2009; Goodson et al. 2015; Hanahan and Weinberg 2000, 2011) together with consideration of environmental chemicals (Nahta et al. 2015), to be sufficiently comprehensive, and accommodate different cancer theories in a pragmatic manner, for chemical hazard assessment purposes. This was also needed to further direct the development of an OECD NGTxC assay database and the systematic evaluation of the relevance and readiness of the assays to enter the TGP. The purpose of this paper is to explain the detailed processes undertaken to develop an IATA for NGTxC that could be acceptable for OECD test guideline development purposes.

## Methodology for developing ranking parameters for the evaluation of the NGTxC-relevant endpoint (in vitro) assays

The methodology for the development of assay ranking parameters was based upon the practical experiences derived from four previous OECD activities [(1) the development and application of OECD thyroid assay scoping document (TSD (OECD 2014b)), (2) OECD GD211 (OECD 2014a), (3) ranking parameters and quantitative scoring system developed by the Developmental Neurotoxicity (DNT) IATA expert group (Bal-Price et al. 2018), (4) activities of the OECD Validation Management Group-Non Animal (VMG-NA) related to test guidelines (e.g. OECD TG 455 (OECD 2016c), and TG 458 (OECD 2016d)), together with (5) form development by the EU Reference Laboratory European Centre for the Validation of Alternative Methods (EURL ECVAM 2019) Test Pre-submission form (EURL ECVAM 2019)].

The ranking parameters and rather extensive quantitative scoring system developed by the DNT IATA expert group (Bal-Price et al. 2018), which also draws on the formats followed for both the TSD (OECD 2014b) and the GD211 (OECD 2014a), were considered, but on the basis of previous experience of expert working groups scoring broad/large amounts of information and the greater number of in vitro*/*ex vivo assays (100 +) to be reviewed (compared to 18 or so for the thyroid and DNT), a more pragmatic and efficient approach as compared to that agreed by the DNT IATA expert group was agreed. The proposed parameters were further discussed, subsequently slightly reorganized and then agreed as part of the outcome of the third face-to-face meeting.

The expert group also agreed that, within the 13 blocks (Identification of assays needed), prioritized according to the general IATA, the assays could be categorized on the basis of scientific merit and development stage into three levels, reflecting their current potential for inclusion in the OECD Test Guidelines work plan, those are as follows:

**Level A—in vitro/ex vivo assays that are ready for validation in the short term**, i.e. could be proposed for OECD Test Guideline development, or are adopted TGs (A+).

**Level B—in vitro/ex vivo assays that could be developed for potential validation in the long term, i.e. after an optimization step**. In addition, assays which meet criteria for Level A but which screen for non-genotoxic carcinogenic mechanism/mode of action endpoints that can be (indirectly) captured through other Level A assays, so would not be prioritized.

**Level C – assays available, but there is not enough information for evaluation and therefore this constitutes an information gap** (no in vitro*/*ex vivo assays identified to cover a specific mode of action or disrupting pathways).

Additional considerations are given below where pertinent to the parameter, to be given where immediately available, but are not essential to that parameter (Table 1). For many of the assays, there is a lack of information on most of the parameters, and further modifications may be necessary, as the assays are reviewed.

**Table 1:** Specific considerations regarding the analysis of individual parameters for NGTxC-relevant assays

Category 1: Initial high-priority considerations		
	Parameter	Description
1	NGTXC Endpoint addressed and intended purpose of the assay	The practical application of the test method (e.g. regulatory/non-regulatory application) Additional consideration: Can the assay be used alone to address the specified endpoint, or should it be used in combination with other methods? e.g. epigenetic assays, metabolism/CYP induction assays (such as addition of S9, etc.)
2	Biological Plausibility	Mechanistic relevance of the test method (e.g. the mechanism of action and its relation to the effect of interest, MIE, KE, KER, etc.)
3	Extrapolation to humans	The assay should use a model system where the response is relevant to those observed in the human system (Additional consideration: Also include information on relevance of this assay to ecotoxicology.)
4	Reference chemicals	Reference chemicals, including positive and negative controls, are required to demonstrate the accuracy and performance of the assay (e.g. “the closeness of agreement between a test method and an accepted reference value’) with respect to the particular molecular mechanism/biological effect being probed. Reference chemicals should be well characterized, covering the relevant applicability domain of the test method, covering a range of structural diversity, be documented for their activity and be readily available. They should be representative of the chemical classes and potencies, including sufficient number of negatives, for which the assay is expected to be used. Ideally, all reference chemicals should be commercially available
5	Availability of a detailed protocol / SOP	The assay method should be described in sufficient detail to allow effective replication
6	Within-laboratory reproducibility	Reproducibility of results within a single laboratory over time, using a defined protocol and the same laboratory set-up A determination of the extent that qualified people within the same laboratory can successfully replicate results using a specific protocol at different times. Also referred to as intra-laboratory reproducibility (OECD 2014b)

Category 2: assay performance considerations		
	Parameter	Description
7	Between-laboratory reproducibility	A measure of the extent to which different qualified laboratories, using the same protocol and testing the same substances, can produce qualitatively and quantitatively similar results. Between-laboratory reproducibility indicates the extent to which a test method can be successfully transferred between laboratories, also referred to as inter- laboratory reproducibility
8	Characterization of assay variability	This parameter demonstrates the degree of variability in the replicates for an assay as expressed as the standard deviations (SD) or coefficient of variation (CVs). E.g. A response readout with excessively high CV would be unacceptable
9	Accuracy	This parameter is about assessing if a high level of concordance exists between the results from the in vitro assay and the result expected for reference chemicals, e.g. from a specific mode of action, in vivo, clinical or epidemiological data When assessing accuracy, one should consider uncertainty in the reference data. Any known uncertainties of the reference data should be captured and considered in the definition of a meaningful benchmark, to be met or exceeded by the new methods Reasonable concordance with reference data needs to be evident
10	Assay specificity	This parameter assesses the rate of true negative results and rate of false positive results when testing the accepted reference chemicals. The ratio of true positive samples over the total number of samples that give positive results (true positives + false negatives) should be provided if available When assessing specificity, one should consider uncertainty in the reference data. Reasonable concordance with reference data needs to be evident
11	Assay sensitivity	This parameter assesses the rate of true positive results and rate of false negatives when testing the accepted reference chemicals. The ratio of the true negative samples over the total number of samples that give negative results (true negatives + false positives) should be provided if available. When assessing sensitivity, one should consider uncertainty in the reference data. Reasonable concordance with reference data needs to be evident
12	Consideration of confounding factors	Attributes of the assay that minimize or detect the presence of confounding factors, and/or reduce the occurrence of inconclusive or incorrect measurements and other bias, e.g. cell toxicity shall be assessed in order to reliably demonstrate an antagonistic activity and avoid confusion due to increased cell toxicity, chemical luminescence or fluorescence that interferes with response signals Availability of quality and acceptance criteria
13	Data interpretation and prediction model	Availability of a procedure for deriving, on the basis of the raw data, the test method endpoint (i.e. biological effect) results
14	Limitation of the test method	This can include limitations identified through testing and/ or cell line characterization (e.g. chemical categories for which the test method does not make reliable and/ or relevant predictions), technical limitations (e.g. not applicable for the testing of poorly soluble materials), and/or mechanistic limitations in relation to known modes of action, and experimental model (e.g. cell line component interference)

Category 3: technical capabilities for test methods		
	Parameter	Description
15	Dynamic range	This parameter considers the degree of change of the response readout from the assay. It should be sufficiently robust to allow sensitive detection of increasing dosage of active substances
16	Concentration test range	This parameter assesses the degree to which features, inherent in the model system, may limit the dose range of substance that can be tested (i.e. need for water solubility, low tolerance for common vehicles, etc.)
17	Response characterization	The readout of the assay should generate a statistically significant change when an effective concentration of active substance is tested

Category 4: other practical considerations		
	Parameter	Description
18	Availability of the assay and essential components	Availability or possibility to transfer the assay to another laboratory (The expertise and technology required for the performance of the assay should be easily acquired or widely available. Ideally, the assay should not be based on complex, highly expensive, technically challenging platforms. However, as expertise and technology can evolve rapidly, this should not be considered a limiting factor.)
19	Intellectual Property rights	This assesses whether any component of the test method (e.g. protocol, test system, equipment) is licensed, patented, copyright protected, trade-marketed, registered or treated as confidential business information (CBI) Needs to be identified whether or not the holder of the patent does/does not support assay development for TG purposes
20	Cost of the assay and essential components	Other limitations that might hinder the ability to acquire the assay components should be assessed. E.g. Current licensing fees
21	Through-put of the assay	Whether low, medium or high throughput, or can be adapted to higher throughput
22	Documentation of development and utility of the method	Any additional information from proof-of-concept or pre-validation exercise (s) not provided in the previous parameters, but relevant, should be available

The ranking parameters are tiered and defined as follows:

### Category 1: high priority: initial considerations

The parameters in this category are considered of highest priority. In addition, each parameter within this category is considered to have equal weight and all are essential for an acceptable assay, i.e. a poor rating on any one is considered to severely impair the validation or regulatory acceptance of the assay.

### Category 2: high priority: assay performance

These parameters relate to the reliability and predictive capacity of the assay itself. Generally, these parameters would have high priority in considerations of the potential for the development of a protocol for a candidate assay to be developed into an OECD Test Guideline. All 8 parameters within this category are considered to have equal weight.

### Category 3: technical capabilities

The parameters in this category also relate to assay performance but the particular performance issues considered under this category of parameters were identified to be of lesser significance compared to the Category 2 performance issues.

### Category 4: other practical considerations

This category lists parameters which may present some challenges to validation or broad acceptance of the protocol as an OECD Test Guideline but are not insurmountable. Consequently, these were identified as being of lowest priority. All of the parameters in this list are of equal importance.

## Ranking

The ranking of ‘**Strong’ (A), ‘Moderate’ (B), ‘Weak’ (C),** for each parameter, was conducted as a group together, to assure consistency amongst the expert group, and then the work progressed as subgroups to split the workload into more manageable pieces, whilst also ensuring balance in expertise and sector, with no one reviewing assays that they have developed in-house. Test methods that are fully validated, and are approved TGs are A + and although they do not need to be assessed for suitability to enter the TGP, as they have already succeeded, there may be need to adapt the qualitative data interpretation models, to ensure suitability for IATA use, beyond that of first level/tier screening, for prioritization purposes, to understand the critical concentrations necessary to move to the next step on the pathway in the IATA.

**Fig. 1:**
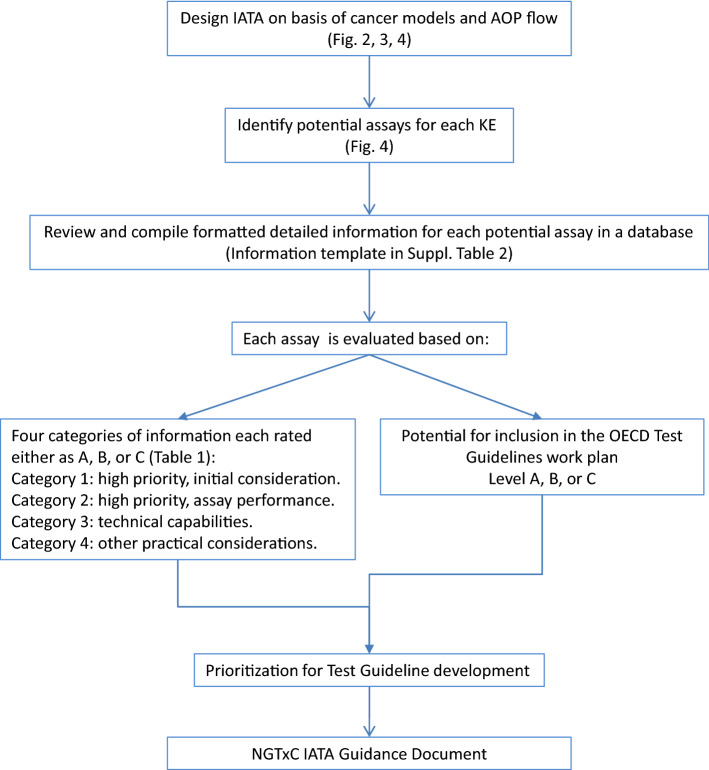
Steps undertaken in the development of the NGTxC IATA and assay evaluations

A summary overview of the steps being undertaken in the development of the NGTxC IATA and (in vitro) assay evaluations addressing the IATA key event blocks is shown in Fig. 1.

The detail on the methodology employed for the collection of the assays to enter the database was not part of the third meeting of the expert group and will be described in the follow-up report on the evaluation of NGTxC assays. Briefly, as systematic review approaches generated an extensive number of inappropriate hits, more targeted approaches included searching assay databases, approaching assay developers and publishing a call to assay developers.

Additional assays with demonstrated performance for the detection of NGTxC can still be provided to the OECD expert working group, over the next year, using the template available in Supplementary Information Table 2).

## Development of an initial AOP framework

To start developing the IATA, different simplified mechanistic, organ-specific cancer pathway development models were built, i.e. liver, colon and lung (Jacobs et al. 2016).

These models exemplify the natural history of the tumour onset and progression for some cancers that share common characteristics in humans. These models are generalized and include a non-exhaustive list of molecular events which contribute to tumorigenesis, whether alone or in combination with other events (Arpino et al. 2009; Espinoza et al. 2016; Giuliano et al. 2011; Kanthan et al. 2012; Sakamaki et al. 2017; Sun et al. 2016; Tariq and Ghias 2016; Villanueva 2019; Yu and Schwabe 2017). On the basis of general cancer pathways, we have used the models to pinpoint where specific cancer hallmarks can be assigned to a specific stage of the disease. In this way, we can better understand where the cancer hallmarks belong to each NGTxC KE. In addition, they can help to develop a basis for identifying test methods able to address endpoints that would be appropriate for using as the biochemical and morphological anchoring of molecular alterations sustaining the point of no return, leading to carcinogenesis and changes in tissue morphology.

**Fig. 2:**
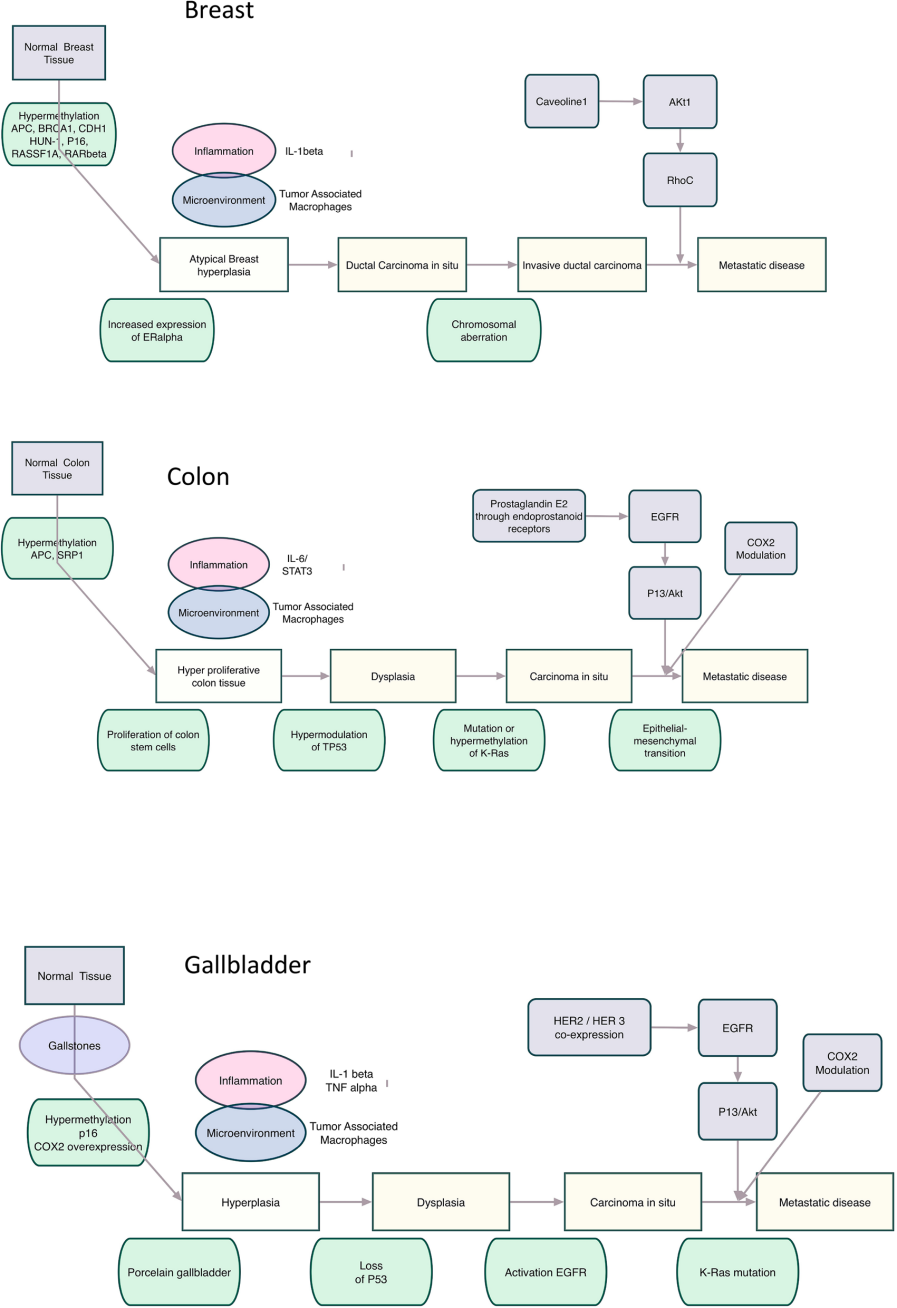
Simplified comminality models of the natural history of cancer for colon, breast and gall bladder exemplifying common key events. Simplified pathways for modelling the natural history of cancer to exemplify critical common stages of key events for use as a basis to derive an overarching commonality IATA. Examples given for colon, breast and gall bladder cancers, with colour and shape coded boxes and text to draw out the commonalities (Reference examples include and are not limited to: (Arpino et al. 2009; Espinoza et al. 2016; Giuliano et al. 2011; Kanthan et al. 2012; Sakamaki et al. 2017; Sun et al. 2016; Tariq and Ghias 2016; Villanueva 2019; Yu and Schwabe 2017)

The colon cancer NGTxC model has also been used as a case study to demonstrate common key events between the colon, breast and gall bladder cancer (Fig. 2).

Similar common mechanistic elements were drawn out from the models previously published (Jacobs et al. 2016) to be utilized as a starting point for a simple, pragmatic NGTxC flow using the Adverse Outcome Pathway (AOP) construct, rather than the more complex organ-specific natural history of cancer models shown in Fig. 2.

With this flow, starting from the Molecular Initiating Event (MIE), defined as the initial point of chemical–biological interaction within the organism that starts the pathway (OECD 2017a), a specific concentration and duration of exposure would be sufficient to reach a threshold at which the next key event, such as inflammation and immune dysfunction, could occur (BOX 3 “Inflammation and immune dependent inflammation as KE common across cancers”). Then at this step, again the tipping point would occur when sufficient genome anomalies would lead to atypical cellular proliferation (dysplastic change). (Metaplasia is considered an adaptive injury related response, and one of the possible sustaining morphological changes consequent to chronic inflammation or chronic stimulation, and it does not progress.)

For the MIE and these early key events, whilst they may be necessary, they might not be sufficient to lead to a cancer outcome. The subsequent key event of a dysplastic change in morphology, which is the defining step essential for tumour formation, would similarly have resulted from the threshold/tipping point being reached in the precursor key event. A couple of caveats include the observation that the events might not necessarily be sequential but may happen concurrently. Also, with respect to the downstream signalling pathways following the MIE, the chemical triggering the MIE may not be interacting directly with the subsequent mechanisms or hallmarks. Rather, these may be triggered upstream by specific biological signalling molecules and biological pathways that have been induced by (sustained) activation of the MIE. This does also mean that well-characterized and well-understood assays that incorporate more of the essential key events (KEs), including the MIE, together with pivotal downstream KEs will be of greater utility to the IATA than assays focusing on individual KEs.

## Box 3. Inflammation and immune-dependent inflammation as KE common across cancers

With respect to the inflammation KE, it was noted that chronic inflammation is not always essential for proliferation, and it does not always lead to tumour formation, but that it has various roles and is a contributory mechanism for the development of cancer. Signs of inflammation can, therefore, be considered as early and sensitive indicators for an increased cancer risk under many real-life conditions (Colotta et al. 2009; Schetter et al. 2010; Zuo et al. 2014), where additional stressors may also come into play (see also BOX 4 “Uncertainties of the current rodent cancer bioassay-based approach to carcinogenicity assessment”). Indeed when the inflammation response is immune mediated, there is a dual immune role: the initial protective defence mechanism via immunosurveillance response (THC-1, interferon gamma, IL 12 and IL 23, all of which participate in the inflammasome) may be replaced by immune evasion (IL 1 beta, IL 6, TNF, (Kravchenko et al. 2015) depending upon the type of immune proteins that are produced (Biswas and Mantovani 2010). This latter immune role can contribute to the development of cancer.

It is particularly noteworthy that as yet there are no standardized or mandatory test methods or OECD TGs that include functional inflammatory parameters.

**Fig. 3:**
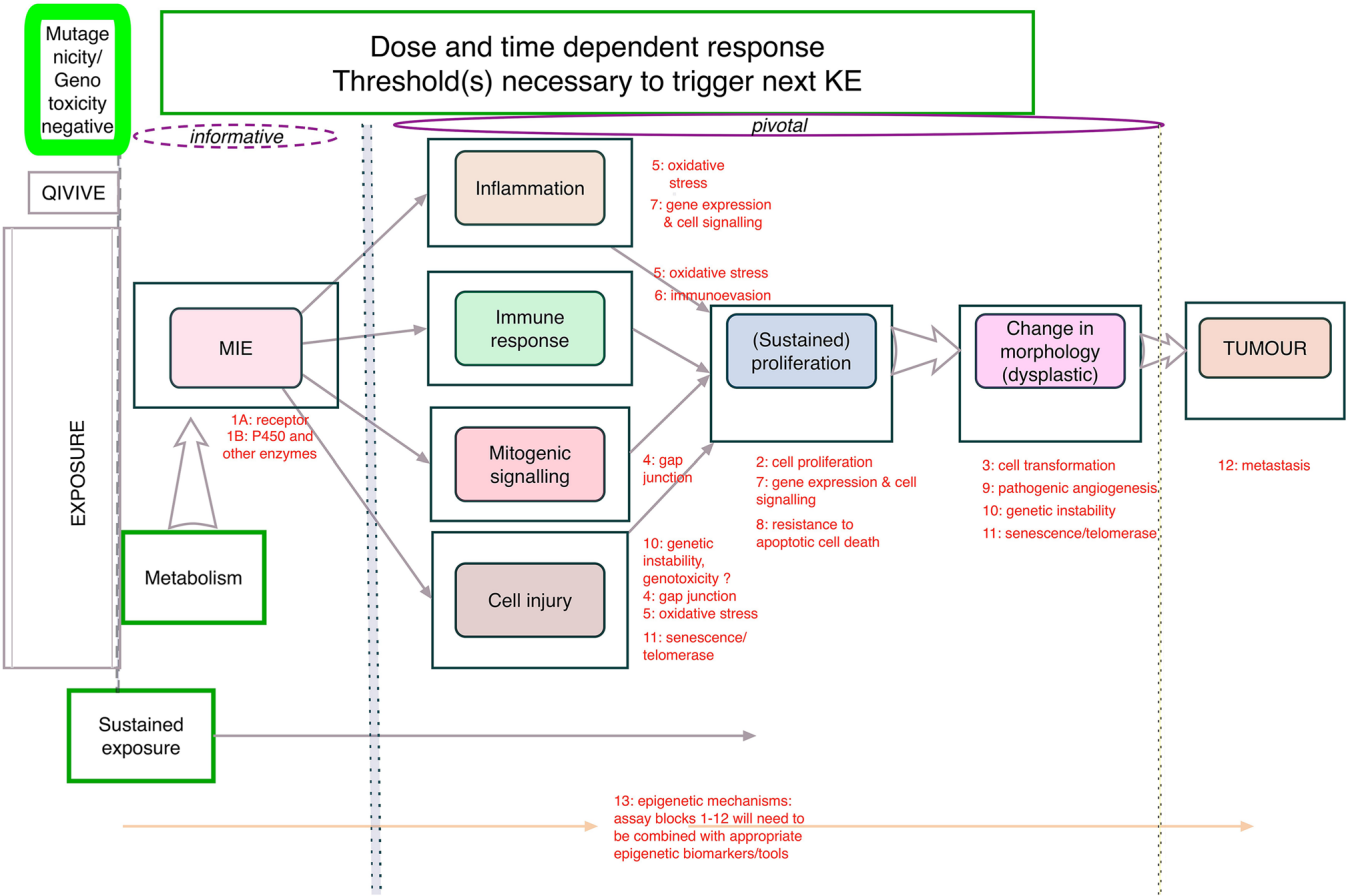
A general integrated approach for the testing and assessment of non -genotoxic carcinogens. The first step of a cancer endpoint hazard assessment is to conduct mutagenicity and genotoxicity testing (top far left hand thick green framed box), for which there are already well established in vitro and in vivo testing paradigms in regulatory toxicology. Considerations in relation to metabolism, exposure and quantitative in vitro and in vivo extrapolations (QIVIVE) would contribute to the overall risk assessment, as indicated on the far left. Sustained exposure is a critical consideration throughout all the modules of the IATA, as this is likely to trigger subsequent modules. Substances that are negative for mutagenicity and genotoxicity would enter the NGTxC IATA, particularly screening, for the cascade of downstream key events for which there are several suitable assays including some validated assays and TGs. Each module sits within a box frame that will be populated by relevant assays, including epigenetic and cofactor assay components, as many of the modules may be subjected to epigenetic deregulation known to be influential in modulating the specific hallmark module. Bound by broken lines on both the left and right-hand sides, central to the IATA, are six pivotal modules, of which four are not consequent or sequential to each other and can lead to (sustained) proliferation. These are as follows: inflammation (including assays that address the hallmark blocks covering oxidative stress and gene and cell signalling); immune response (again including assays for oxidative stress, but also immune evasion assay models, as they mark the passage /turning point from the body’s immune defence to the immune evasion by the tumour); mitotic signalling (including assays addressing the gap junction hallmark); and finally cell injury (including assays addressing the hallmarks of genetic instability, gap junction, oxidative stress and senescence and telomerase). The fifth module is (sustained) proliferation, and here the essential assay hallmark to be addressed is cell proliferation, triggering investigations on gene and cell signalling and resistance to apoptotic cell death. The sixth module, a change in morphology (dysplastic change), represents the point at which adaptive (sustained) proliferation, -hyperplasia becomes mal-adaptive. The change in morphology module also includes early key events of cell transdifferentiation (at the cellular level that is conversion of one differentiated cell type into another cell type), such as changes in the organization of the cytoskeleton, acquisition of different morphology) and progression to mal-adaptive/irreversible modifications, specifically pathogenic angiogenesis (in contrast to neoangiogenesis which could be adaptive modifications), genetic instability, and then senescence and telomerase activation. The seventh and final module is the tumour stage that is addressed by the metastasis cancer hallmark

**Fig. 4:**
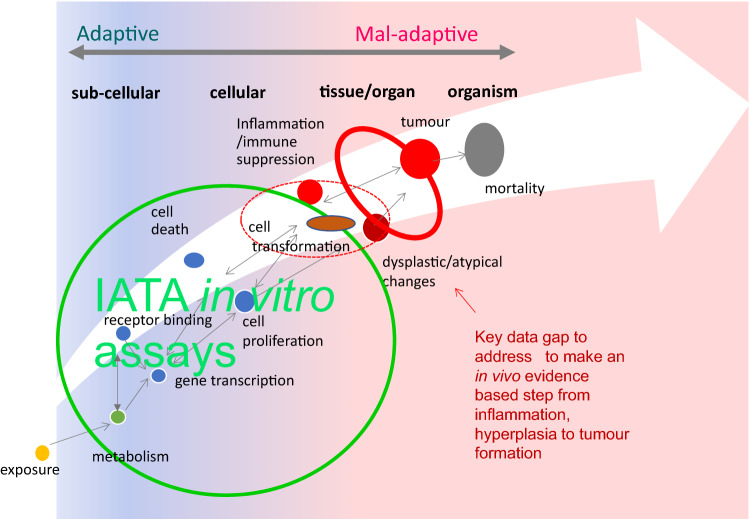
Conceptual overview of the adaptive versus mal-adaptive critical data gaps for adverse outcome recognition in NGTxC. From adaptive to mal-adaptive disease progression: key data gaps in the testing and assessment of non-genotoxic carcinogenicity (adapted from Paparella et al. 2016). There are numerous in vitro assays to address the early key events from receptor binding and transactivation, gene transcription, metabolism and cell proliferation (indicated by the green circle on the left of the figure). Assays are also available for cell transformation, both for early (initiation) and later (promotion) phases (broken red line elipse). A change in morphology represents the point at which adaptive (sustained) proliferation and hyperplasia/dysplasia become mal-adaptive, and this is the key data gap to make the in vivo evidence-based step from hyperplasia to tumour formation (solid red lined elipse). This tipping point is histopathologically characterized with cellular and/or structural atypia. This change is often observed as abnormal nuclear division and disorganized cell proliferation with loss of cell polarity; therefore, in vitro assays that can be used to explore and test these aspects are of high priority

It is important to note that NGTxCs may also act by mechanisms independent of inflammatory pathways (as indicated in Figs 2, 3, 4), and absence of an inflammatory response is not a guarantee of safety. Some, but not all inflammatory response aspects are currently followed in toxicity testing programmes.

In the pharmaceutical sector, usually the standard toxicity studies are considered sufficient to assess potential immunosuppression for pharmaceuticals in animals (ICH 2005a, b). Signs that are taken into account include the following:

1. Hematological changes such as leukocytopenia/leukocytosis, granulocytopenia/granulocytosis, or lymphopenia/lymphocytosis;
2. Significant alterations in immune system organ weights and/or histology (e.g. changes in thymus, spleen, lymph nodes and/or bone marrow);
3. Changes in serum globulins that occur without a plausible explanation, such as effects on the liver or kidney, these can be an indication that there are changes in serum immunoglobulins;
4. Increased incidence of infections, which may suggest immunosuppression.

Pivotal studies will subsequently be conducted in humans, where the signs above are observed.

For pharmaceuticals, human data are available for points 1, 3 and 4.

The T cell-dependent antibody response (TDAR) assay used in nonclinical studies of pharmaceutical chemicals is also used with other toxicology assessments to assess immune system function that is dependent upon the effectiveness of multiple immune processes, including antigen uptake and presentation, T cell, B cell activation and antibody production (ICH 2005a, b; Lebrec et al. 2014).

Currently, in the agrochemical sectors, immunopathology and humoral immunity (including assessment of immune suppression according to OPPTS guidelines; US EPA, 1996), in short-term studies, may be used for screening purposes. However, following retrospective analysis, the utility of this screening as a ‘standard approach’ for all chemicals has been questioned by the same agency, for this chemical space (US EPA 2013). In addition, the National Toxicology Programme (NTP) study protocols (these are not internationally recognized test guidelines) utilize a testing battery including standard toxicity testing endpoints as well as cell-mediated (proliferative responses) and non-specific immunity (NK cell assay), whilst the non-standard aspects include immunophenotyping (also included in the extended one generation reproductive toxicity TG, OECD TG 443 (OECD 2018c)), humoral and cell-mediated immunity and host resistance assays (Luster et al. 1988, 1992). In the EU, in practice, agrochemical registration dossiers rarely contain this information, perhaps because harmonized test methods do not specifically request it, or with respect to the immunotoxicity cohorts in TG 443, these are rarely requested or provided.

Within the framework of the IATA for NGTxC, it is noted that some of the scientific literature claims that most immunosuppressive agents are not positive in the RCB. However, the data do not support that. For example, for two very old studies, tacrolimus (FK506) and cyclosporine, systemic bioavailability was exceedingly low, such that the studies in rodents should be considered inadequate, and gavage should have been used. Subsequent studies of FK506 applied topically showed lymphomas in the RCB in under two years (Contrera et al. 1995). Whilst there are examples of chemicals with non-genotoxic carcinogenic modes of action in rats that are not relevant to humans, this does not include immunosuppressants.

It is also recognized that disease- or drug-induced immunosuppression may contribute in certain cases to human carcinogenesis, for example causing decreased surveillance to infectious organisms (such as EBV, HPV, and HHV8) or other factors which may increase cancer risk in immunocompromised and transplant patients (NIH 2015) or being co-contributor to cancer development in cases where chronic inflammation is a contributing factor (as described by Axelrad and colleagues for bowel cancer (Axelrad et al. 2016)). If chemically induced immunosuppression co-occurs at levels relevant to other mechanisms of carcinogenesis, such as chronic inflammation, then the potential for it to contribute to carcinogenesis should be considered in a weight of evidence approach within the context of this IATA.

An IATA that includes a more thorough hazard assessment of the immune dysfunction, including a respective point of departure, would be valuable to contribute to the prevention of several systemic toxicities, including cancer. In current regulatory requirements/decision-making, only immunosuppression is characterized (in some sectors, under certain conditions). Indication of inflammation may be detected by histopathology, but the presence of infiltrates is associated with tumorigenesis only for certain tumour types (i.e. cytotoxicity/inflammation followed by regenerative response), but this is less the case for other mechanisms (such as receptor-mediated toxicity, for example).

## Configuration of the IATA

The work of this expert group is specifically targeted to carcinogenicity hazard characterization and therefore further specific KEs in addition to immunosuppression also need to be considered. The preliminary NGTxC flow discussion model proved to be an appropriate simple prototype to facilitate the discussions that led to the development of the overarching NGTxC IATA (Fig. 3).

Generally, the first step of a cancer endpoint hazard assessment is to conduct mutagenicity and genotoxicity testing (top left hand green framed box, Fig. 3), for which there are already well-established in vitro and in vivo testing paradigms in regulatory toxicology. A substance that is positive for mutagenicity can immediately be classified as a mutagen according to the UN GHS category 2 classification for mutagenicity and/ or Class 1 carcinogen (e.g. for IARC and the US EPA), discussed in Jacobs et al. (2016).

Considerations in relation to exposure and quantitative in vitro and in vivo extrapolations (QIVIVE) would contribute to the overall risk assessment, as indicated on the far left of the Fig. 3. However, sustained exposure is a critical consideration throughout all the modules of the IATA, as this is likely to trigger subsequent modules. Substances that are negative for mutagenicity and genotoxicity would enter the NGTxC IATA, particularly screening for the cascade of downstream key events for which there are several suitable assays including some validated assays and TGs. Each module sits within a box frame that will be populated by relevant assays, including epigenetic and cofactor assay components, as many of the modules may be subjected to epigenetic deregulation known to be influential in modulating the specific hallmark module.

There are four pivotal modules in the IATA that are not consequent or sequential to each other and can lead to (sustained) proliferation (Fig. 3). These are as follows: inflammation (including assays that address the hallmark blocks covering oxidative stress and gene and cell signalling); immune response (again including assays for oxidative stress, but also immune evasion assay models, as they mark the passage /turning point from the body’s immune defence to the immune evasion by the tumour); mitotic signalling (including assays addressing the gap junction hallmark); and finally cell injury (including assays addressing the hallmarks of genetic instability, gap junction, oxidative stress and senescence and telomerase). Again, several suitable assays are available for these modules (see Table 2).

The next step is (sustained) proliferation, and here the essential assay hallmark to be addressed is cell proliferation, triggering investigations on gene and cell signalling and resistance to apoptotic cell death.

A change in morphology (dysplastic change) represents the point at which adaptive (sustained) proliferation and hyperplasia become mal-adaptive.

This tipping point is histopathologically characterized with cellular and/or structural atypia. This change is often observed as abnormal mitosis and disorganized cell proliferation with loss of cell polarity. At the moment, the interpretation of histological findings to predict carcinogenicity is currently based on the most conservative approach, of observing benign tumours and carcinomas. However, there are distinct differences in the molecular biology of cancer between rodent and human particularly with respect to timing and control of tumour growth and progression (Goodman and Wilson 1991; Holliday 1996; Wolf et al. 2019). Ultimately, the IATA needs to address the complexity of human biological processes of cancer development.

The change in morphology module also includes early key events of cell transdifferentiation (at the cellular level that is conversion of one differentiated cell type into another cell type), such as changes in the organization of the cytoskeleton, acquisition of different morphology and progression to mal-adaptive/irreversible modifications, specifically pathogenic angiogenesis (in contrast to neoangiogenesis which could be adaptive modifications), genetic instability, and then senescence and telomerase activation.

With the recent characterization work conducted for the CTAs, it can now be shown that the in vitro assays that can address some of these hallmarks include the validated BALB/c 3T3 CTA (EURL ECVAM 2012), the SHE CTA (Corvi et al. 2012; EURL ECVAM 2012), and the Bhas 42 CTA (EURL ECVAM 2012; OECD 2017b). The CTAs are able to highlight various stages from early to late cell transformation (OECD 2017b; Serra et al. 2019). For the Bhas 42 CTA, the initial step is Ras activation, whilst the promotion protocol can add further information, with repeated exposure promoting cell proliferation and following focus formation (Ohmori et al. 2004). There are also a very few suitable in vitro assay models for the other hallmarks identified, but overall, these appear to be less well characterized. A further in vitro assay/data gap that is important for the in vitro regulatory toxicology approach is to make the initial in vivo evidence-based step from the earlier modules to those of hyperplasia, dysplasia and tumour formation (Fig. 4). Whilst the (process of) acquisition of invasion and metastasis cancer hallmark is an essential biological characteristic of malignant tumours, by this stage it may or may not be related to chemical exposure, and is a lower priority for regulatory purposes.

## Key considerations

There are a number of fundamental considerations that need accommodation as the IATA is built and populated with appropriate assays that will necessarily evolve over time. As the IATA is intended to protect human health, ideally the IATA should be based on cells and tissues that best mimic the human in vivo situation. At the current time, however, such cell systems are not sufficiently well developed for broad regulatory use; therefore, a more pragmatic approach is being undertaken to facilitate the use of the assay tools in the near future, as well as encouraging the more ‘ideal’ assay tools to be developed. Therefore, for practical reasons, the key events addressed in the IATA are likely to include other mammalian cell systems, and, in the more immediate future, will also likely require in vivo tests (e.g. 3–7 day, 28 and 90-day assays) and tests such as the TDAR discussed in the preceding section, to address mid to late key events. Despite species differences, in vivo assay information has utility in clarifying the sequence of early events and identifying early cancer key event markers contributing to cancer development that cannot always be easily obtained from human clinical transcriptomics data, as the disease is usually already established in human clinical observation studies. The IATA will not, however, include the rodent cancer bioassay (as explained in BOX 4 “Uncertainties of the current rodent cancer bioassay-based approach to carcinogenicity assessment”). These key considerations are discussed here and below, in more detail.

## Box 4. Uncertainties of the current rodent cancer bioassay-based approach to carcinogenicity assessment

It is not appropriate to consider the current rodent cancer bioassay**-**based approach as a ‘gold standard’. Its uncertainties have been comprehensively characterized (Paparella et al. 2016) using an evaluation grid (template) that was originally designed for the reporting of defined in vitro approaches for testing and assessment (OECD 2016b). This repurposing of the OECD template for in vivo test characterization shall facilitate a future comparative uncertainty assessment with the new IATA, where the performance of the current rodent cancer bioassay-based approach shall be the benchmark for the minimum performance of the new IATA. This comprehensive uncertainty characterization is important because correlation of alternative assessment results with reference animal results is limited by the uncertainties and complexity of the latter. Ultimately, this approach is intended to support the validation of future IATA needs based upon an integration of various data streams, including essential basic science knowledge on cancer AOPs/MoAs, and utilizing clinical and epidemiological cancer data (where available and of sufficient power) to support the KEs. Under constantly changing real-life conditions, various (epi) genetic backgrounds, pre-existing disease states and stages, co-exposure and additional environmental stressors may come into play. Therefore, in vivo test endpoints (as for in vitro test endpoints) cannot be easily judged as absolutely “adverse” or “not adverse” effects. Their contribution to tumour development depends on the variable real-life conditions and environments, which cannot be comprehensively tested for regulatory purposes either in vivo or in vitro. Consequently, future IATAs may include molecular and cellular endpoints that provide early and sensitive indicators for an increased cancer risk, based upon the relative potency within a MoA, among tested chemicals. The publication introducing this perspective also suggests that with a longer-term view, this may become the basis for the development of a new in vitro MoA hazard class including potency differentiation (Paparella et al. 2016).

## Key considerations: Duration of exposure

Generally, for NGTxC related tumour formation, the duration of exposure is an important factor to be considered, since tumour formation in vivo is almost always associated with sustained exposure. The concentration, length and timing of exposure needed to trigger the next step in tumour formation are worthy of close scrutiny.

If exposure stops within a certain timeframe, at specific time points before the tipping or threshold point prior to an atypical change in proliferation or tissue morphology, the process towards adversity may be halted and the extent of damage may decrease with no or reduced exposure, depending on the specific real-life situation, where other stressors come into play (e.g. (Gray et al. 2017; Parsons et al. 2010; Robsahm et al. 2019; Sakamaki et al. 2017).

There is a need to better understand the conditions, the key molecular changes and concentrations that may lead to the continuation of the adversity process. And to do this, where available, information on the concentration response relationships is being collected for the assays in the OECD NGTxC database.^2^ Also, metabolism and kinetic quantitative in vitro to in vivo extrapolation (QIVIVE) modelling will be necessary to appropriately interpret in vitro concentrations.

## Key considerations: Distinguishing between factors that lead to neoplasms, versus those that lead to hyperplasia

It is important to be able to distinguish between factors that lead to neoplasms versus factors that only lead to hyperplasia, and no further, so the expert group will need to revisit this consideration. In addition, if the simplified flow is called an AOP, it needs to include the adverse outcome at organism level and should thus include tumour formation. For current work, however, it was agreed to first concentrate efforts on the central modules of the IATA (immune/inflammatory/mitotic/cell injury responses and proliferation), rather than on the modules for initiating molecular events and tumour/metastasis formation. Discussions regarding how the adverse event could be defined are very much ongoing. Whilst some views have expressed concern that an atypical change in cell morphology would be seen as an adverse outcome with the construct in Fig. 3, within the uncertainty approach being applied, other views expressed were that molecular and cellular key events may be considered as early, sensitive indicators for toxicity and indeed collectively these adaptations may become the basis of a new concept of adversity. It is intended to design the NGTxC IATA toolbox of assays such that one will be able to discern whether the cascade of events is likely to stop at the morphology change or whether it is likely to be moving towards the adverse apical outcome, i.e. tumour formation. For example, where subsequent to a cell morphology change, modifications in the cell cytoskeleton occur, such that the result is more than simply a change in cell morphology, assays that can highlight the nature of the cell cytoskeleton reorganization (Cirillo et al. 2017) and similarities to the dysplasic characteristics seen in human cancer are useful [e.g.(Kanaan et al. 2010; Mascolo et al. 2018; OECD 2017b)].

## Key considerations: The IATA in the short term: supporting the transition to an in vitro IATA

The MoA of a chemical substance contributing to the development of carcinogenesis can be species-specific, and this is extensively discussed in sister publications (Jacobs et al. 2016; Paparella et al. 2016) and many others (e.g. (Marone et al. 2014; Meek et al. 2014; Thayer and Foster 2007)). Although regulation of chemicals should protect humans and wildlife, ultimately the goal is to agree on human-relevant mode of actions a priori to testing and thus only include mechanisms of human relevance into the IATA. Such an approach might be difficult to achieve in short term, as current core regulatory testing regimes are based upon intact animal models.

‘Omics tools, particularly transcriptomics, are of great utility in supporting the transition, in different ways (Chakraborty et al. 2018; Occhipinti et al. 2020; Schaap et al. 2015). For example, the use of transcriptomics information to identify key markers of cancer in humans will relate to the more advanced steps and late stages of cancer clinically identified, whilst transcriptomics in vitro will generally relate to the first steps in cancer development (i.e. identifying and characterizing the early mechanistic steps in both the in vitro assays and human cancers, as early diagnostic markers of disease); this means correlating in vitro data with human clinical cancer ‘omics data may not be straight forward with respect to determining early biomarkers of cancer development, for the NGTxC IATA purposes. Thus, the question of timing and/or possible critical windows in cancer development cannot be solved solely by human clinical research. It seems that in the transition period to an in vitro-based IATA, a more inclusive approach would include relevant NGTxC biomarkers. In the short term, the inclusion of in vivo studies would (1) better satisfy current chemical testing paradigms, (2) increase identification of relevant markers and (3) improve the evidence base for in vitro assay development for a truly in vitro NGTxC IATA. Therefore, during the first review of assays, it was necessary to consider different species-specific MoA (not only those already fully agreed as human relevant).

## Population of the overarching IATA framework with relevant assays

### Identification of assays needed

The cancer hallmarks (Goodson et al. 2015; Hanahan and Weinberg 2000, 2011), as adapted in Jacobs et al. (2016), have been used to provide a grouping format for organizing relevant assays to address the biological processes associated with cancer initiation, promotion/progression and tumour formation for regulatory test method development IATA purposes. A number of test methods that provide information on these early molecular initiating steps/ very early key events, and later key events that are available from the public literature and assay databases, are being collated and evaluated. Table 2 provides the key hallmarks of NGTxC (further exemplification can be found in Supplementary Information Table 1) and representative standardized (or commonly used) tests that address them.

**Table 2:** Key hallmarks of NGTxC and representative international standardized tests, that can address these hallmarks

Block	Key hallmarks /characteristics (Mechanism/ mode of action)	IATA modules	Readiness level/currently used in hazard assessment^a^	Selected examples of representative standardized (*or commonly used*) assays
1a	Receptor binding and activation also as part of hormone-mediated processes	MIE		
	Testing for receptor binding		A: Adopted as OECD, EU and US EPA TG	Oestrogen Receptor (ER) Binding Assay OECD TG 493 US EPA 890.1250
	Testing for receptor agonism/antagonism		A: Adopted as OECD, EU and US EPA TG	Oestrogen receptor (ER) transactivation assays OECD TG 455 US EPA 890.1300
			A: Adopted as OECD TG	Androgen receptor transactivation assay OECD TG 458
			B: Undergoing ISO validation 2019-current	Aryl hydrocarbon receptor (AhR) transactivation assay, US EPA METHOD 4435 (2008); JIS K 0463 (2009)(Aarts et al. 1995; Garrison et al. 1996; Han et al. 2004)
	Alteration of hormone synthesis		A: Adopted as OECD and US EPA TG	Steroidogenesis H295R Assay OECD TG 456 US EPA 890.1550
			A: Adopted as US EPA OCSPP HTG^b^	Aromatase US EPA 890.1200
1b	CYP P450 induction	MIE	B: On OECD TGP workplan: validated, draft OECD TG under consideration	HepRG® CYP induction test method (Bernasconi et al. 2019)
2	Cell proliferation	(Sustained) proliferation, mitogenic signalling	No in vitro TGs, no in vitro assays on the OECD TGP workplan	Repeated Dose 90-Day Oral Toxicity Study in Rodents OECD TG 408, US EPA 870.3100
			A: Adopted as OECD and US EPA TG In vivo	
			*Test methods currently used to contribute to regulatory WoE assessment*	*Cell proliferation* in vivo (*Ki-67*) (Jouinot and Bertherat 2018) (Wood et al. 2015) *Hepatic DNA synthesis and cell proliferation* (US FDA 2018; Wood et al. 2015) *Bromodeoxyuridine (BrdU) assays* in vivo
3	Cell transformation	Change in morphology	A: Adopted as OECD and US EPA TG	TG 408 US EPA 870.3100 90 day in vivo histopathological features
			A: ICH guideline	ICH S6 PART 1(ICH 2011a, b; Wang et al. 2010)
			B: Validated assays not adopted as OECD TG; OECD GD (OECD 2015, 2017b)	Bhas 42 Cell Transformation Assay (OECD 2017b)
				Syrian Hamster Embryo (SHE) Cell Transformation Assay(OECD 2015)
				BALB/c 3T3 Transformation Assay
4	Gap junction intercellular communication	Change in morphology, mitogenic signalling	No TGs, no assays on the OECD TGP workplan	
5	Indicators of oxidative stress	Inflammation, immune response, Cell injury	B: Assays in (pre) validation and on the OECD TGP workplan	Reactive Oxygen Species (ROS) generation assay (phototoxicity)
				Toxtracker (Hendriks et al. 2012, 2016)
			*C: Test methods currently used to contribute to regulatory WoE assessment*	*HepG2 peroxide formation*
				*Malondialdehyde (MDA)—Thiobarbituric Acid Reactive Substances (TBARs)* ex vivo *assays*
				*in vivo assays, ICH* (ICH 2005a, b)
6	Immunosuppression/ evasion	Immune response	A: ICH S8 (ICH 2005a, b) and OECD TGs (TG 443)	NK cell/Host resistance and others
			A: ICH S8(ICH 2005a, b)	TDAR- immunosuppression tier 1 screening
			A: TG 443(OECD 2018c) US EPA, OPPTS^c^ 870.7800 Immunotoxicity. (1996) US EPA, OCSPP Test Guideline No. 870. 7800). (2013)	
7	Gene expression and cell signalling pathways	Inflammation, immune response, mitogenic signalling	B: Assays in (pre) validation and on the OECD TGP workplan	Toxtracker (Henriks et al. 2012, 2016)
8	Increased resistance to apoptotic cell death	Cell injury	A: OECD TG 408	Histopathology from TG 408 90 days studies (standard picnotic nuclei)
				Histopathology from single or repeated dose studies (eg TG 408 90 day studies): Use of special stains on ex vivo Tissue slices Hematoxilin/Eosin staining Fluoro-jade staining
				*Caspase 3/7 activation* in vivo
				*DNA fragmentation*
9	Pathogenic angiogenesis and neoangiogenesis	Change in morphology	No TGs, no assays on the OECD TGP workplan	
10	Genetic Instability (Disturbed DNA repair, quick establishment of mutations = “mutator phenotype”)	Change in morphology Cell injury	No TGs, no assays on the OECD TGP workplan	
11	Cellular senescence/ telomerase	Change in morphology, Cell injury	No TGs, no assays on the OECD TGP workplan	
12	Metastasis (migration, intra- and extravasation), survival outside of original tissue	Tumour	No TGs, no assays on the OECD TGP workplan	
13	Epigenetic mechanisms and associated genetic instability	All	No TGs, no assays on the OECD TGP workplan	(Greally and Jacobs 2013) reviews how TGs could be augmented

^a^TG readiness levels A, B, C are described in the methodology section

^b^OCSPP HTG: US EPA Office of Chemical Safety and Pollution Prevention Harmonized Test Guidelines

^c^OPPTS: US EPA Office of Prevention, Pesticides and Toxic Substances

The information on the assays was initially structured in a similar way to that conducted for the OECD Thyroid Scoping Document (OECD 2014b), where a page summary is provided for each of the relevant mechanistic and MoA assays, in a consistent reporting manner to enable cross comparison and prioritization for readiness to be developed and validated as an OECD TG. Following the publication of the OECD Guidance Document for describing non-guideline in vitro methods (OECD 2014a), the format has been modified accordingly. The database therefore provides the critical information requirements on a broad sweep of in vitro assays for the key events that could be considered as a basis for an IATA of NGTxC. It is organized into thirteen cancer hallmark assay blocks that address early to mid to later key events with consequent increasing associations to adversity.

These include the following key mechanisms/modes of action: receptor binding and activation also as part of hormone-mediated processes, cell proliferation, cell transformation, gap junction intercellular communication, CYP P450 induction, induction of oxidative stress, immunosuppression/immune evasion, gene expression and cell signalling pathways, increased resistance to apoptotic cell death, pathogenic angiogenesis and neoangiogenesis, epigenetic mechanisms and genetic instability, cellular senescence/ telomerase, and invasion and metastasis (migration, intra- and extravasation, survival outside of original tissue) (Jacobs et al. 2016).

Common test methods currently used for assessment of potential of target pharmacology and weight of evidence evaluation of toxicity data to support a mechanistic argument, in registration dossiers (but that are not necessarily officially standardized test methods), are indicated in italics.

Although the database focuses primarily in vitro assays, we cannot ignore the current regulatory environment which relies on in vivo animal information. Therefore, also pragmatic in vivo solutions for use in the short term are being investigated. In current practice, for sectors such as agrochemicals, pharmaceuticals and biocides, we should make better use of well-identified early cancer key event markers discernible from the information generated from existing in vivo TGs, such as routine 28- and 90- day studies, but also other short-term in vivo studies, such as a 26-week study with Ras H2 transgenic mice (as has been conducted for pharmaceuticals). The use of transgenic animals in sectors other than pharma has been criticized from an animal use perspective, since it would require animal intensive validation; therefore, the investment in non-animal approaches, in line with the 3Rs, is preferred. Identifying relevant early key cancer biomarkers in the short-term in vivo studies requires careful discussion and consensus. For example, Ki-67 is a well-understood clinically derived marker of the acceleration of cell proliferation that has also been examined in short-term studies (Jouinot and Bertherat 2018; Wood et al. 2015). However, proliferation by itself is not a sufficient condition to imply carcinogenesis risk and in many cases may represent an expected physiologic adaptive or therapeutic response for some targeted pharmaceuticals (e.g. induction of erythroid hyperplasia in bone marrow by erythropoietin-stimulating therapies for anaemia). Indeed, our IATA work will need to reconcile this balanced accuracy issue, providing adequate sensitivity whilst still discriminating adverse signals from those that are incidental or adaptive.

Using a systematic review approach combined with assay database mining, supplemented with expert knowledge from the group, an OECD call for assays, and invitations to the audiences at scientific meetings, overall more than 100 in vitro assays are identified so far, within these 13 blocks, with varying degrees of literature confidence with respect to assay reliability, sensitivity, efficiency, technical criteria and chemical applicability domain. They are currently undergoing evaluation by the group according to the criteria shown in Table 1. Additional work is underway with respect to populating this database, to better identify the mechanism and assay gaps, but also with respect to expert review of the assays for toxicological relevance of the target sites, chemical space interrogated by the assay and consequently assessment of readiness for validation in the short, medium and long term.

Combinations of these assays into the IATA are likely to allow discrimination between non-adverse and adverse changes occurring in humans/in vivo mammalian test systems following activation of MIE/early KE. They will represent additional lines of evidence within IATA, ideally indicating strong biological relevance among the predictive in vitro findings (Fig. 5 adapted from (Paparella et al. 2016)).

## Population of the assay database: Gaps identified

In the process of populating the database, specific preliminary in vitro assay gaps were identified, particularly those that involve the atypical alteration of the cell/tissue morphology giving rise to the irreversible autonomous neoplasia step, which, representing the point of no return, is a crucial step in the tumorigenesis process. The gaps were further elucidated in a call for assays announced by the OECD early in 2018. Specifically, in vitro assays that address the following human-relevant cancer biomarkers are still needed:

1. Immune response and inflammatory biomarkers, and specifically measurement of cytokine release (e.g. IL 6: as it has a pivotal role in the acquisition of the malignant phenotype)
2. Cytoskeleton modification, specifically to understand the link between cytoskeleton changes and the link with carcinogenic processes; therefore, assays that assess cytoskeleton modification are needed to discriminate between adaptive to the adverse response (Butcher et al. 2009; Cirillo et al. 2017; Holth et al. 1998).
3. Cancer specific kinase activation

Also, a preference for the following test systems was expressed:

i. Carcinogenesis-relevant cell models that can highlight morphological and behavioural changes that can be used for phenotypic anchoring of human-relevant carcinogenic steps and inflammatory biomarkers
ii. 3D models to address the complexity at the tissue level that the individual cell-based molecular-based assays cannot address

Tests that highlight the progression to transformation are particularly essential. To this end, the acquired transformed characteristics can be confirmed using cell models that have the ability to grow in soft agar, have high chemotactic ability and have the capacity to cross barriers and impact upon the loss of adhesion molecules (*e.g.* soft agar assay, chemotaxis assay, Matrigel assay), and therefore, the call extended also to such assays.

Experience from the pharmaceutical sector with respect to biomarkers of cancer therapy is also of high value. For example, assays/endpoints/biomarkers of carcinogenic disease such as Rb2/p130 (retinoblastoma protein 2, a key antiapoptotic factor highly active in many cancer types (Pentimalli et al. 2018)), and kinase activation have already been successfully used within the pharmaceutical sector, in the development of chemotherapeutics and in cancer therapy. These endpoints and/or assays highlight biomarkers of cancer therapy and are, due to their key role in sustaining the tumour progression, of fundamental importance for the identification of possible non-genotoxic carcinogens.

Additional suggestions for reference substances, which are known carcinogens but negative in common genotoxicity tests, were also requested, together with a request for the assays to be able to examine potency/concentration response.

To collect comprehensive information on relevant test methods in a harmonized way, a template was developed in accordance with OECD guidance on non-guideline test methods description (OECD 2014a) and is available as supplementary information (Supplementary Information Table 2).

## Discussion and forward look

Here, we have described the challenging task of developing a complex IATA scheme for NGTxC and our main recommendations (summarized in BOX 5 “Summary of main recommendations and outputs of the third meeting of the OECD expert working group developing an IATA for NGTxC”), based upon commonalties in the natural history of human cancer models. We have also shown the methodology that we are employing to evaluate and prioritize appropriate, mainly in vitro assays to address the respective key events.

Thus far, we have identified several in vitro and in vivo assays that are already addressing MIEs and are also TGs (e.g. TG 455 (OECD 2016c), TG 458 (OECD 2016d) and we are in the process of evaluating many further (non-standardized) assays.

## Box 5. Summary of main recommendations and outputs of the third meeting of the OECD expert working group developing an IATA for NGTxC

The NGTxC IATA:

- Has a working definition for harmonized regulatory purposes,
- Is designed to accommodate all OECD regulatory jurisdictions,
- Will meet regulatory confidence for implementation of the selected assays, by undergoing strict specified evaluation and ranking criteria,
- Should include assays that address the pivotal key mechanistic events of:
- inflammation,
- immune response,
- mitogenic signalling,
- cell injury
- leading to (sustained) proliferation and a dysplastic change in morphology,
- and will need to be combined with appropriate epigenetic biomarkers/tools.

Having agreed this consensus statement and developed the IATA framework and evaluation criteria for assays to populate the IATA we will next be able to populate the IATA assay tool box and prioritize assay development.

In addition to the parameters described in Table 1, the prioritization of assays should consider refinement in data interpretation and whenever applicable, origin of antibodies, preferably non-animal (Viegas Barroso et al. 2020). Concentration response data for each of the assays will be useful for understanding the thresholds that are associated with positive results for the various assays across the IATA. Also, within the relevant TGs that are available, in addition to the dichotomous categorization of positives and negatives answers, we will also need information for point of departure for hazard characterization and limit value derivation, which requires more quantitative data interpretation tools (e.g. integrating data variability in point of departures such as benchmark doses, NOAEL, LOAEL etc.) within the TGs. Exploiting the wealth of concentration–response information contained in the assay data has also been recognized during the peer review of in vitro test method validation efforts as critical input for approaches towards quantitative hazard and risk assessment, such as IATA (EURL ECVAM 2020).

 As noted at the outset, the value of the current rodent cancer bioassay to identify the hazards of potential carcinogens has been highly and extensively criticized. To reduce or avoid the use of the rodent cancer bioassay, and to benefit from emerging improvements and new replacement assays that can be included in the IATA, the current working group is liaising with ongoing initiatives in the USA and Europe, particularly with respect the hazard relevant initiatives appropriate to the OECD TGP. A “waiver concept” to the rodent cancer bioassay was developed for pharmaceuticals, also called ‘NegCarc’ (Negative for Endocrine, Genotoxicity and Chronic Study Associated Histopathological Risk factors for Carcinogenicity) approach (Alden et al. 2011; Sistare et al. 2011). European Commission and industry efforts ongoing in Europe at the EPAA (European Partnership for Alternative Approaches to Animal testing) level focus on a mechanism-based approach to further improve the predictivity of the NegCarc approach for agrochemicals, using mechanistic information obtained from in silico*, *in vitro and short-term in vivo studies. Similarly, a US Environmental Protection Agency (EPA) and animal welfare non-governmental organization (People for the Ethical Treatment of Animals: PETA International Science Consortium Ltd) task force are also examining how to develop a waiver for the rodent cancer bioassay for agrochemicals, using WoE approaches on a breadth of relevant endpoints used in both hazard and risk assessment-including exposure (Cohen et al. 2019). In addition, the expert group will discuss aspects of the in vivo International Council for Harmonization of Technical Requirements for Pharmaceuticals for Human Use (ICH) S1B carcinogenicity guideline for the testing of pharmaceuticals (ICH 2017, 2019).

These have been proposed as alternatives to the long-term rodent cancer bioassay, particularly where subsequent evaluations have shown the assay(s) to be robust, which is the case for the in vivo transgenic Hras2 mouse model, for example (Jacobs and Brown 2015). Of particular interest to Japan is the medium-term (8 week) rat liver carcinogenesis assay, which has been shown to detect genotoxic and non-genotoxic hepatocarcinogens with over 90% sensitivity and overall known carcinogens with about 60% sensitivity (Ito et al. 2003).

In the shorter term, the first version of the assay integrated NGTxC IATA will need to include these current practices using in vivo diagnostic tools and (augmented) test method approaches, and so is unlikely to be completely in vitro based. It is highly likely that recommendations will be made to include additional parameters, e.g. inflammation and immune markers in the shorter (3–7 day), 28 and 90-day studies. As the evidence base builds over time, this can evolve such that the long-term goal is to ultimately achieve a fully in vitro human-relevant NGTxC IATA that is appropriate and acceptable for global regulatory needs.

Further optimization of the IATA will require the identification of what is "sufficient" information for categorizing chemicals as carcinogens. This may need to look at weighting the various assay blocks in a different manner, for example, it may not be necessary to ‘prove’ metastasis steps but stop at the point where adequate prediction can be achieved to the satisfaction of stakeholders, including industry, regulatory bodies and non-governmental organizations. With respect to chemicals identified as immunosuppressive agents, it may not be necessary to run the chemical also in a cell transformation assay, as such substances will not directly transform cells but will decrease immunosurveillance of carcinogenic infectious organisms. We will then apply the uncertainty analysis approach specifically developed for the assay evaluation and IATA development having assessed and ranked the assays in the database, and together with weight of evidence assessment, the next steps will be to refine the IATA and develop decision trees, together with case study testing of the IATA.

Selected assays will then be subject to OECD review to arrive at a final guidance document suitable for the development and (pre)validation of the priority in vitro tests for the human-relevant IATA, such that it will be clear which tests are appropriate for specific KEs. Funding initiatives will then be able to target selected assay validation for TG use within the IATA.

## Electronic supplementary material

Below is the link to the electronic supplementary material.

Supplementary file1 Supplementary Table 1. (separate document: Table 1, Major mechanisms of non-genotoxic carcinogenicity, and suggested organization for IATA development, updated from Jacobs et al., 2016) (DOCX 149 kb)

Supplementary file2 Supplementary Table 2. Template for assay information for inclusion in the NGTxC assay database (XLSX 20 kb)

## Abbreviations

AR: Androgen receptor
AOP: Adverse outcome pathway
BMD: Benchmark dose
CTA: Cell transformation assay
CYP P450: Cytochrome P450
EPAA: European Partnership for Alternative Approaches to Animal testing
IATA: Integrated Approach to Testing and Assessment
IARC: International Agency for Research on Cancer
ICH: International Council for Harmonization of Technical Requirements for Pharmaceuticals for Human Use
KE: Key event
KER: Key event relationship
GD: Guidance document
GHS: Globally Harmonized System
MAD: Mutual Acceptance of Data
MIE: Molecular initiating event
MoA: Mode of action
NGTx: Non-genotoxic
NGTxC: Non-genotoxic carcinogen(s)
NTP: National Toxicology Programme
OECD: Organization for Economic Cooperation and Development
QIVIVE: Quantitative in vitro to in vivo extrapolation
(Q)SAR: (Quantitative) Structure Activity Relationship
TDAR: T cell-dependent antibody response assay
TG: Test guideline
TSD: Thyroid assay scoping document
US EPA: US Environmental Protection Agency
WoE: Weight of Evidence

## Footnotes

1 It is noted that not all genotoxic events lead to mutagenicity, and that some prefer the terminology “mutagenic mode of action”. However, genotoxicity assays are still commonly used to distinguish those chemicals with the potential to directly affect the integrity of DNA from those that do not, so for the sake of simplicity, the text throughout refers to genotoxic versus non-genotoxic carcinogens.

2 This database is still under construction and evaluation, and it will become part of the publicly available OECD NGTxC IATA Guidance Document once completed.

## References

[CR1] Aarts JM, Denison MS, Cox MA (1995). Species-specific antagonism of Ah receptor action by 2,2',5,5'-tetrachloro- and 2,2',3,3'4,4'-hexachlorobiphenyl. Eur J Pharmacol.

[CR2] Adler S, Basketter D, Creton S (2011). Alternative (non-animal) methods for cosmetics testing: current status and future prospects-2010. Arch Toxicol.

[CR3] Alden CL, Lynn A, Bourdeau A (2011). A critical review of the effectiveness of rodent pharmaceutical carcinogenesis testing in predicting for human risk. Vet Pathol.

[CR4] Ankley GT, Bennett RS, Erickson RJ (2010). Adverse outcome pathways: a conceptual framework to support ecotoxicology research and risk assessment. Environ Toxicol Chem.

[CR5] Arpino G, De Angelis C, Giuliano M (2009). Molecular mechanism and clinical implications of endocrine therapy resistance in breast cancer. Oncology.

[CR6] Axelrad JE, Lichtiger S, Yajnik V (2016). Inflammatory bowel disease and cancer: the role of inflammation, immunosuppression, and cancer treatment. World J Gastroenterol.

[CR7] Bal-Price A, Hogberg HT, Crofton KM (2018). Recommendation on test readiness criteria for new approach methods in toxicology: exemplified for developmental neurotoxicity. Altex.

[CR8] Bernasconi C, Pelkonen O, Andersson TB (2019). Validation of in vitro methods for human cytochrome P450 enzyme induction: Outcome of a multi-laboratory study. Toxicol In Vitro.

[CR9] Biswas SK, Mantovani A (2010). Macrophage plasticity and interaction with lymphocyte subsets: cancer as a paradigm. Nat Immunol.

[CR10] Boobis AR, Cohen SM, Dellarco VL (2016). Classification schemes for carcinogenicity based on hazard-identification have become outmoded and serve neither science nor society. Regulat Toxicol Pharmacol.

[CR11] Boobis AR, Cohen SM, Doerrer NG (2009). A data-based assessment of alternative strategies for identification of potential human cancer hazards. Toxicol Pathol.

[CR12] Butcher DT, Alliston T, Weaver VM (2009). A tense situation: forcing tumour progression. Nat Rev Cancer.

[CR13] Chakraborty S, Hosen MI, Ahmed M, Shekhar HU (2018). Onco-Multi-OMICS approach: a new frontier in cancer research. Biomed Res Int.

[CR14] Cirillo L, Gotta M, Meraldi P (2017). The elephant in the room: the role of microtubules in cancer. Adv Exp Med Biol.

[CR15] Cohen SM (2004). Human carcinogenic risk evaluation: an alternative approach to the two-year rodent bioassay. Toxicol Sci.

[CR16] Cohen SM (2010). An enhanced 13-week bioassay: an alternative to the 2-year bioassay to screen for human carcinogenesis. Exp Toxicol Pathol.

[CR17] Cohen SM (2010). Evaluation of possible carcinogenic risk to humans based on liver tumors in rodent assays: the two-year bioassay is no longer necessary. Toxicol Pathol.

[CR18] Cohen SM (2018). Screening for human urinary bladder carcinogens: two-year bioassay is unnecessary. Toxicol Res.

[CR19] Cohen SM, Boobis AR, Dellarco VL (2019). Chemical carcinogenicity revisited 3: Risk assessment of carcinogenic potential based on the current state of knowledge of carcinogenesis in humans. Regulat Toxicol Pharmacol.

[CR20] Colotta F, Allavena P, Sica A, Garlanda C, Mantovani A (2009). Cancer-related inflammation, the seventh hallmark of cancer: links to genetic instability. Carcinogenesis.

[CR21] Contrera JF, Jacobs AC, Prasanna HR, Mehta M, Schmidt WJ, de George J (1995). A systemic exposure-based alternative to the maximum tolerated dose for carcinogenicity studies of human therapeutics. J Am Coll Toxicol.

[CR22] Corvi R, Aardema MJ, Gribaldo L (2012). ECVAM prevalidation study on in vitro cell transformation assays: general outline and conclusions of the study. Mutat Res.

[CR24] EURL ECVAM (2012) Recommendation concerning the cell transformation assays (CTA) using Syrian Hamster Embryo cells (SHE) and the BALB/c 3T3 mouse fibroblast cell line for in vitro carcinogenicity testing, including the ESAC opinion (Annex 1) based on the ESAC peer review of an EURL ECVAM-coordinated validation study of three CTA protocols for in vitro carcinogenicity testing https://ihcp.jrc.ec.europa.eu/our_labs/eurl-ecvam/eurl-ecvamrecommendations/cta-recommendation

[CR23] EURL ECVAM (2019) Test pre-submission form. eu science hub: test method submission. (https://ec.europa.eu/jrc/en/eurl/ecvam/alternative-methods-toxicitytesting/validation/test-method-submission). Accessed 10 Sep 2019

[CR26] EURL ECVAM (2020) EU Reference Laboratory European Commission Validation of Alternative Methods (EURL ECVAM) Scientific Advisory Committee (ESAC) peer review opinion on the AR CALUX™ test method (in press)

[CR27] Espinoza JA, Bizama C, Garcia P (2016). The inflammatory inception of gallbladder cancer. Biochem Biophys Acta.

[CR28] Garrison PM, Tullis K, Aarts JM, Brouwer A, Giesy JP, Denison MS (1996). Species-specific recombinant cell lines as bioassay systems for the detection of 2,3,7,8-tetrachlorodibenzo-p-dioxin-like chemicals. Fundament Appl Toxicol.

[CR29] Giuliano M, Schifp R, Osborne CK, Trivedi MV (2011). Biological mechanisms and clinical implications of endocrine resistance in breast cancer. Breast (Edinburgh, Scotland).

[CR30] Goodman G, Wilson R (1991). Predicting the carcinogenicity of chemicals in humans from rodent bioassay data. Environ Health Perspect.

[CR31] Goodman JI (2018). Goodbye to the bioassay. Toxicol Res.

[CR32] Goodson WH, Lowe L, Carpenter DO (2015). Assessing the carcinogenic potential of low-dose exposures to chemical mixtures in the environment: the challenge ahead. Carcinogenesis.

[CR33] Gottmann E, Kramer S, Pfahringer B, Helma C (2001). Data quality in predictive toxicology: reproducibility of rodent carcinogenicity experiments. Environ Health Perspect.

[CR34] Gray A, Grushchak S, Mudaliar K, Kliethermes S, Carey K, Hutchens KA (2017). The microenvironment in primary cutaneous melanoma with associated spontaneous tumor regression: evaluation for T-regulatory cells and the presence of an immunosuppressive microenvironment. Melanoma Res.

[CR35] Greally JM, Jacobs MN (2013). In vitro and in vivo testing methods of epigenomic endpoints for evaluating endocrine disruptors. Altex.

[CR36] Han D, Nagy SR, Denison MS (2004). Comparison of recombinant cell bioassays for the detection of Ah receptor agonists. BioFactors (Oxford, England).

[CR37] Hanahan D, Weinberg RA (2000). The hallmarks of cancer. Cell.

[CR38] Hanahan D, Weinberg RA (2011). Hallmarks of cancer: the next generation. Cell.

[CR39] Hendriks G, Atallah M, Morolli B (2012). The ToxTracker assay: novel GFP reporter systems that provide mechanistic insight into the genotoxic properties of chemicals. Toxicol Sci.

[CR40] Hendriks G, Derr RS, Misovic B, Morolli B, Calleja FM, Vrieling H (2016). The extended toxtracker assay discriminates between induction of dna damage, oxidative stress, and protein misfolding. Toxicol Sci.

[CR41] Holliday R (1996). Neoplastic transformation: the contrasting stability of human and mouse cells. Cancer Surv.

[CR42] Holth LT, Chadee DN, Spencer VA, Samuel SK, Safneck JR, Davie JR (1998). Chromatin, nuclear matrix and the cytoskeleton: role of cell structure in neoplastic transformation (review). Int J Oncol.

[CR43] ICH (2005a) International Conference on Harmonisation of technical requirements for registration of pharmaceuticals for human use. 10.1111/j.1365-2125.1994.tb05705.x PMC1364893 8054244

[CR44] ICH (2005b) harmonised tripartite guideline. Immunotoxicity studies for human pharmaceuticals, S8 Current Step 4 version.https://www.ich.org/fileadmin/Public_Web_Site/ICH_Products/Guidelines/Safety/S8/Step4/S8_Guideline.pdf Accessed 11 July 2019.

[CR45] ICH (2011a) ICH S6 (R1) Preclinical safety evaluation of biotechnology-derived pharmaceuticals. 10.1038/nrd822 12119749

[CR46] ICH (2011b) International Conference on Harmonisation ICH S6 (R1) Preclinical safety evaluation of biotechnology-derived pharmaceuticals 22616137

[CR47] ICH (2017) International Conference on Harmonisation of technical requirements for registration of pharmaceuticals for human use. The ICH Regulatory Testing Paradigm of Carcinogenicity in Rats. Status report 2017. https://www.ich.org/fileadmin/Public_Web_Site/ICH_Products/Guidelines/Safety/S1/S1_Status_Report_PEP_2018_0207.pdf. Accessed 11 July 2019.

[CR48] ICH (2019) International Conference on Harmonisation of technical requirements for registration of pharmaceuticals for human use. The ICHS1 Regulatory Testing Paradigm of Carcinogenicity in rats. Status Report 2019. https://www.ich.org/fileadmin/Public_Web_Site/ICH_Products/Guidelines/Safety/S1/S1_Status_Report_PEP_2018_0207.pdf. Accessed 2 Sep 2019.

[CR49] Ito N, Tamano S, Shirai T (2003). A medium-term rat liver bioassay for rapid in vivo detection of carcinogenic potential of chemicals. Cancer Sci.

[CR50] Jacobs AC, Brown PC (2015). Regulatory forum opinion piece*: transgenic/alternative carcinogenicity assays: a retrospective review of studies submitted to CDER/FDA 1997–2014. Toxicol Pathol.

[CR51] Jacobs MN, Colacci A, Louekari K (2016). International regulatory needs for development of an IATA for non-genotoxic carcinogenic chemical substances. Altex.

[CR52] JIS K 0463 (2009) Japanese industrial standard: guidelines for reporter gene assay binding on aryl hydrocarbon receptor-Assay of dioxins in an Ah Receptor.

[CR53] Jouinot A, Bertherat J (2018). Management of endocrine disease: adrenocortical carcinoma: differentiating the good from the poor prognosis tumors. Eur J Endocrinol.

[CR54] Kanaan Z, Qadan M, Eichenberger MR, Galandiuk S (2010). The actin-cytoskeleton pathway and its potential role in inflammatory bowel disease-associated human colorectal cancer. Genet Test Mol Biomarkers.

[CR55] Kanthan R, Senger JL, Kanthan SC (2012). Molecular events in primary and metastatic colorectal carcinoma: a review. Pathol Res Int.

[CR56] Kravchenko J, Corsini E, Williams MA (2015). Chemical compounds from anthropogenic environment and immune evasion mechanisms: potential interactions. Carcinogenesis.

[CR57] Lebrec H, Molinier B, Boverhof D (2014). The T-cell-dependent antibody response assay in nonclinical studies of pharmaceuticals and chemicals: study design, data analysis, interpretation. Regulat Toxicol Pharmacol.

[CR58] Luijten M, Olthof ED, Hakkert BC (2016). An integrative test strategy for cancer hazard identification. Crit Rev Toxicol.

[CR59] Luster MI, Munson AE, Thomas PT (1988). Development of a testing battery to assess chemical-induced immunotoxicity: National Toxicology Program's guidelines for immunotoxicity evaluation in mice. Fundament Appl Toxicol.

[CR60] Luster MI, Portier C, Pait DG (1992). Risk assessment in immunotoxicology I. Sensitivity and predictability of immune tests. Fundament Appl Toxicol.

[CR61] Madia F, Worth A, Whelan M, Corvi R (2019). Carcinogenicity assessment: addressing the challenges of cancer and chemicals in the environment. Environ Int.

[CR62] Madia F, Worth A, Corvi R (2016). Analysis of carcinogenicity testing for regulatory purposes in the European Union.

[CR63] Marone PA, Hall WC, Hayes AW (2014). Reassessing the two-year rodent carcinogenicity bioassay: a review of the applicability to human risk and current perspectives. Regulat Toxicol Pharmacol.

[CR64] Mascolo MG, Perdichizzi S, Vaccari M (2018). The transformics assay: first steps for the development of an integrated approach to investigate the malignant cell transformation in vitro. Carcinogenesis.

[CR65] Meek ME, Boobis A, Cote I (2014). New developments in the evolution and application of the WHO/IPCS framework on mode of action/species concordance analysis. J Appl Toxicol.

[CR66] Nahta R, Al-Mulla F, Al-Temaimi R (2015). Mechanisms of environmental chemicals that enable the cancer hallmark of evasion of growth suppression. Carcinogenesis.

[CR67] NIH, 2015. National Cancer institute, Cancer Causes and Prevention, Immunosuppression. 29 Apr 2015, Available at https://www.cancer.gov/about-cancer/causes-prevention/risk/immunosuppression

[CR68] Occhipinti A, Hamadi Y, Kugler H, Wintersteiger C, Yordanov B, Angione C (2020). Discovering essential multiple gene effects through large scale optimization: an application to human cancer metabolism. IEEE/ACM Trans Comput Biol Bioinf.

[CR71] OECD (2014a) Guidance document for describing non-guideline in vitro test methods. Series on testing and assessment No. 211 OECD, Parishttp://www.oecd.org/officialdocuments/publicdisplaydocumentpdf/?cote=ENV/JM/MONO(2014)35&doclanguage=en

[CR72] OECD (2014). New Scoping Document on In Vitro and Ex Vivo Assays for the Identification of Modulators of Thyroid Hormone Signalling: Series on Testing and Assessment No 207. OECD, Paris.

[CR73] OECD (2015) Guidance Document on the in vitro syrian hamster embryo (SHE) Cell Transformation Assay. . Series on Testing and Assessment 214. OECD, Paris. ENV/JM/MONO(2015)18. https://www.oecd.org/env/ehs/testing/Guidance-Document-on-the-in-vitro-SyrianHamster-Embryo-Cell-Transformation-Assay.pdf.

[CR74] OECD (2016a) Guidance Document for the use of Adverse Outcome Pathways in developing Integrated Approaches to Testing and Assessment Series on Testing and Assessment No. 260, OECD, Paris. http://www.oecd.org/officialdocuments/publicdisplaydocumentpdf/?cote=env/jm/mono(2016)67&doclanguage=en

[CR75] OECD (2016b) Guidance document on the reporting of defined approaches to be used within integrated approaches to testing and assessment. Series on Testing and Assessment No. 255 ENV/JM/MONO (2016). http://www.oecd.org/officialdocuments/publicdisplaydocumentpdf/?cote=env/jm/mono(2016)67&doclanguage=en.

[CR69] OECD (2012). Test No. 455: performance-based test guideline for stably transfected transactivation in vitro assays to detect estrogen receptor agonists.

[CR76] OECD (2016). Test No. 458: stably transfected human androgen receptor transcriptional activation assay for detection of androgenic agonist and antagonist activity of chemicals.

[CR70] OECD (2017a) Guidance document on developing and assessing adverse outcome pathways. Series on testing and assessment No 184. OECD, Paris. http://www.oecd.org/officialdocuments/publicdisplaydocumentpdf/?cote=env/jm/mono(2013)6&doclanguage=en

[CR77] OECD (2017b) Guidance document on the in vitro Bhas 42 cell transformation assay. Series on testing and assessment No. 231. OECD, Parishttp://www.oecd.org/officialdocuments/publicdisplaydocumentpdf/?cote=ENV/JM/MONO(2016)1&doclanguage=en

[CR78] OECD (2018a) Test No. 451: Carcinogenicity studies. OECD, Paris. https://www.oecd-ilibrary.org/environment/test-no-451-carcinogenicity-studies_9789264071186-en

[CR79] OECD (2018b) Test No 453: Combined Chronic Toxicity/Carcinogenicity Studies. OECD, Paris. https://www.oecd-ilibrary.org/environment/test-no-453-combined-chronic-toxicity-carcinogenicity-studies_9789264071223-en

[CR80] OECD (2018). TG 443 extended one-generation reproductive toxicity study.

[CR81] Ohmori K, Sasaki K, Asada S, Tanaka N, Umeda M (2004). An assay method for the prediction of tumor promoting potential of chemicals by the use of Bhas 42 cells. Mutat Res.

[CR82] Paparella M, Colacci A, Jacobs MN (2016). Uncertainties of testing methods: What do we (want to) know about carcinogenicity?. Altex.

[CR83] Parsons A, Daley A, Begh R, Aveyard P (2010). Influence of smoking cessation after diagnosis of early stage lung cancer on prognosis: systematic review of observational studies with meta-analysis. BMJ.

[CR84] Pentimalli F, Forte IM, Esposito L (2018). RBL2/p130 is a direct AKT target and is required to induce apoptosis upon AKT inhibition in lung cancer and mesothelioma cell lines. Oncogene.

[CR85] Robsahm TE, Heir T, Sandvik L (2019). Changes in midlife fitness, body mass index, and smoking influence cancer incidence and mortality: A prospective cohort study in men. Cancer Med.

[CR86] Sakamaki A, Kamimura K, Abe S (2017). Spontaneous regression of hepatocellular carcinoma: a mini-review. World J Gastroenterol.

[CR87] Schaap MM, Wackers PF, Zwart EP (2015). A novel toxicogenomics-based approach to categorize (non-)genotoxic carcinogens. Arch Toxicol.

[CR88] Schetter AJ, Heegaard NH, Harris CC (2010). Inflammation and cancer: interweaving microRNA, free radical, cytokine and p53 pathways. Carcinogenesis.

[CR89] Serra S, Vaccari M, Mascolo MG (2019). Hazard assessment of air pollutants: The transforming ability of complex pollutant mixtures in the Bhas 42 cell model. Altex.

[CR90] Sistare FD, Morton D, Alden C (2011). An analysis of pharmaceutical experience with decades of rat carcinogenicity testing: support for a proposal to modify current regulatory guidelines. Toxicol Pathol.

[CR91] Sonich-Mullin C, Fielder R, Wiltse J (2001). IPCS conceptual framework for evaluating a mode of action for chemical carcinogenesis. Regulat Toxicol Pharmacol.

[CR93] Sun JH, Luo Q, Liu LL, Song GB (2016). Liver cancer stem cell markers: Progression and therapeutic implications. World J Gastroenterol.

[CR94] Tariq K, Ghias K (2016). Colorectal cancer carcinogenesis: a review of mechanisms. Cancer Biol Med.

[CR95] Thayer KA, Foster PM (2007). Workgroup report: National Toxicology Program workshop on Hormonally Induced Reproductive Tumors - Relevance of Rodent Bioassays. Environ Health Perspect.

[CR96] UK Committee on Carcinogenicity of Chemicals in Food (2019) Consumer Products and the Environment (COC) Statement COC/G07 - Version 1.1 Alternatives to the 2-year Bioassay. Committee on Carcinogenicity: statements and guidancehttps://assets.publishing.service.gov.uk/government/uploads/system/uploads/attachment_data/file/803050/G07_Alternatives_to_the_2-year_Bioassay_v1.1.pdf .

[CR97] US EPA (1996) Office of Prevention, Pesticides and Toxic substances - Health Effects Test Guidelines OPPTS 870.7800 Immunotoxicity.

[CR98] US EPA (2013) A Retrospective Analysis of the Immunotoxicity Study (OCSPP Test Guideline No. 7800). https://www.epa.gov/sites/production/files/documents/immunotoxicity-retroanalysis.pdf Accessed 15 July 2019.

[CR99] US FDA (2018) US Food and Drug Administration, Freedom of information Summary, Supplemental Animal Drugs Application. NADA 41–063, NUFLOR- Florfenicol injectable solution, beef and nonlactating dairy cattle; https://animaldrugsatfda.fda.gov/adafda/app/search/public/document/downloadFoi/355 3(2018). Accessed 12 Dec 2019.

[CR25] Viegas Barroso J, Halder M, Whelan M (2020) EURL ECVAM recommendation on non-animal-derived antibodies, EUR 30185 EN, Publications Office of the European Union, Luxembourg. 10.2760/80554. https://ec.europa.eu/jrc/en/publication/eur-scientific-and-technical-research-reports/eurl-ecvam-recommendation-non-animal-derived-antibodies. Accessed 26 May 2020

[CR100] Villanueva A (2019). Hepatocellular carcinoma. N Engl J Med.

[CR101] Wang T, Jacobson-Kram D, Pilaro AM (2010). ICH guidelines: inception, revision, and implications for drug development. Toxicol Sci.

[CR102] WHO (2007) IPCS framework for analysing the relevance of a cancer mode of action for humans and case studies. Harmonization Project Document No 4. https://www.who.int/ipcs/methods/harmonization/areas/cancer_mode.pdf

[CR103] Wittwehr C et al. (2020) JRC report in preparation.

[CR104] Wolf DC, Cohen SM, Boobis AR (2019). Chemical carcinogenicity revisited 1: a unified theory of carcinogenicity based on contemporary knowledge. Regulat Toxicol Pharmacol.

[CR105] Wood CE, Hukkanen RR, Sura R (2015). Scientific and Regulatory Policy Committee (SRPC) review: interpretation and use of cell proliferation data in cancer risk assessment. Toxicol Pathol.

[CR106] Woutersen M, Beekman M, Pronk MEJ, Muller A, de Knecht JA, Hakkert BC (2018). Does REACH provide sufficient information to regulate mutagenic and carcinogenic substances. Human Ecol Risk Assess.

[CR107] Yu LX, Schwabe RF (2017). The gut microbiome and liver cancer: mechanisms and clinical translation. Nat Rev Gastroenterol Hepatol.

[CR108] Zuo H, Tell GS, Vollset SE (2014). Interferon-gamma-induced inflammatory markers and the risk of cancer: the Hordaland Health Study. Cancer.

