# Supplementary material for: Chemical carcinogen safety testing: OECD expert group international consensus on the development of an integrated approach for the testing and assessment of chemical non-genotoxic carcinogens
Source: Arch Toxicol. 2020 Jun 27;94(8):2899–923. doi: 10.1007/s00204-020-02784-5 (PMC7395040; doi:10.1007/s00204-020-02784-5)
Supplement: Supplementary file 1 — Supplementary file1 Supplementary Table 1. (separate document: Table 1, Major mechanisms of non-genotoxic carcinogenicity, and suggested organization for IATA development, updated from Jacobs et al., 2016) (DOCX 149 kb) [file 204_2020_2784_MOESM1_ESM.docx]

**Supplementary Table. 1: Examples of Major mechanisms of Non Genotoxic Carcinogenicity, and suggested data/information organisation for IATA development**

Level 1: Subcellular, basic molecular information: Molecular initiating event (MIE) and of lower priority

Level 2: Cellular, MIE + additional level of cellular complexity: higher priority

Level 3: Multicellular tissue and organ level at which the cellular changes are sufficient to trigger cytoskeletal, tissue and organ changes

Modified and updated from Jacobs et al. 2016.

| **Level of information: Early key events** | **AOP: Assay block: Key mechanism** | **Morphological alteration at the cell tissue level** | **Relevance of key mechanism(s) / endpoints** | **Level of readiness for TG development/AOP inclusion** | **Main relevant pathways and important genes involved: utility of key event relationships (KERs).** | **Related pathways / cross-interactions** | | **Endpoint/hallmark affected cross-reactions** | **Examples of major genes of the pathways involved** | **Prototype substances associated with early key events** | **Prototype substances associated with adverse outcome** |
| --- | --- | --- | --- | --- | --- | --- | --- | --- | --- | --- | --- |
| 1 | Receptor binding and activation also as part of hormone mediated processes | Examples of measurable morphological alterations as a result of receptor activation and consequent cell proliferation: ER, AR, PR: activation of AKT1 pathway: timing-dependent cell proliferation/apoptosis (Sever and Glass 2013)  Modulation of HSP90.  AhR: MAPK pathway, increase of CYP1A1, crosstalk with related receptor pathways. and cell type-dependent proliferation/apoptosis, affects cellular plasticity through cell-cell and extracellular matrix interactions, cytoskeleton reorganization(Larigot et al. 2018) | Angiogenesis receptors such as VEGF would be relevant, as the tyrosine phosphorylation patters and the activation of major downstream signaling pathways are comparable (Nieminen et al. 2014). | Receptor assays available (some validated (OECD, TG 455, 458). Could be used in combination with gene expression profiles for related pathways.  Potential for adaptation of the Steroidogenesis TG 456 to address the KE of aromatase MoA for an aromatase dependent breast cancer AOP.  Combine with OECD TG 473 but leave out the use of metaphase-arresting substances in exposed cells; this could then be used to screen for epigenetic effects. | Wnt/catenin signaling pathway, i.e.:  DNA modifications (cytosine methylation)  miRNA and RNA expression studies  Studies of chromatin components and structure | ER, AR, PPARs, RXR, RAR, VDR, GR, FXR, AhR and ARNT (see e.g.(Schaap et al. 2015))  Also see below re: VEGF | | Gene expression  Metabolic enzyme induction  Genetic instability  Tumor microenvironment  Cell cycle  Invasion and metastasis | AhR: Modulation of HSP (very early KE)(Cox and Miller 2004)  PPARs: β-catenin(Lin et al. 2014) | DES(Gibson and Saunders 2014)  TCDD(Murray et al. 2014) DEHP(Corton et al. 2014) | DES(Gibson and Saunders 2014) TCDD(Murray et al. 2014) DEHP(Corton et al. 2014) |
| 1 | Cell proliferation assay | As above  +  Early KE steps and hyperplasia | A very early downstream KE | Assays available | p13k-AKT signaling pathway | VEGF signaling pathway plus specific relevant miRNA  MAPK signaling pathway | | Cell proliferation  Angiogenesis  DNA repair | Ras-Raf-1-MEK-ERK | BPA(Song et al. 2015) | Nitric oxide(Choudhari et al. 2013) DES(Wong and Walker 2013) |
| 1/2 | Gap junctional intercellular communication | As above  +  Early KE steps | Endpoint: block of dye transfer  Markers: connexin inhibition or decrease of connexin expression | Assays available and in regulatory use(Swierenga and Yamasaki 1992), e.g., clonogenic soft agar assay included in some pharmaceutical regulatory requirements (also a cell proliferation assay) | Gap junction  Possibly (chemical specific) connected with apoptosis and cell morphological transformation | MAPK signaling pathway | | Apoptosis  Differentiation  Tissue homeostasis | Ras-Raf-1-MEK-ERK, connexins | TPA(Skeberdis et al. 2001) |  |
|  |  | As above  +  Early KE steps |  |  |  | Calcium signaling pathway | | Sustained proliferative signaling | PLC, PKC | TPA(Skeberdis et al. 2001) |  |
| 2 | CYP P450 induction | - | P450 futile cycling, i.e. CYP 2E1. Stabilization of 2E1 protein leads to a prolonged burst of ROS production that may result in tissue necrosis, mutation and malignancy. | CYP 2E1 and 2B induction as predictive marker of potential chemical carcinogenesis due to increased reactive oxygen species production  Assays available | Oxidative stress  Hepatic inflammation  Xenobiotic metabolism | NADPH oxidase activity  Oxidative stress | | Deregulated metabolism | Phospho-Akt(Nakanishi et al. 2005) | Ethanol(Ekstrom and Ingelman-Sundberg 1989; Kukielka and Cederbaum 1992; Parke 1994) | Ethanol |
| 2 |  | - | CYP 1A1 AhR mediated transduction | Assays available | Mitochondrial transcription and mitochondrial stress signaling(Biswas et al. 2008) | ER pathway cross talk(Go et al. 2015)  MMP1, MMP2, MMP9  Retinoic acid signaling(Murphy et al. 2004) | | Correlation with migratory processes and metastasis potential | Induction of alterations in gene expression:  Decrease in E-cadherin  Activation of JNK, increased HEF1/NEDD9/CAS-L | Dioxins  Dioxin-like PCBs  PAH in mixtures | Dioxins  Dioxin-like PCBs |
| 2 | Induction of oxidative stress | - | ROS triggered mitochondrial pathway inducing apoptosis(Lucas and Kolodziej 2015) | Assays available  Can be combined with P450 induction and DNA methylation | p53 signaling pathway  Induction of oxidative stress association with regulation gene expression either directly through activation of gene transcriptional pathways or indirectly through hypomethylation  also specific relevant miRNA  ROS plus anti angiogenic stimuli | Mitochondrial malignancy(DeBerardinis and Chandel 2016; Kudryavtseva et al. 2016; Ralph et al. 2010; Senft and Ronai 2016). Mitochondria are master drivers of oxidative stress in the cell, and they sustain the formation of ROS. In addition, independently from the cellular DNA driven processes, the activation of oncogene expression can be sustained by mitochondrial functions. | | MDM family of p53-proteins (p21 and bcl-2) |  | Paraquat(Black et al. 2008; Chang et al. 2013; Yang and Tiffany-Castiglioni 2008) |  |
|  |  | - |  |  |  | AhR | |  | HMOX | PAH(Ferecatu et al. 2010) |  |
|  |  | - |  |  |  | Casp-3-dependent PKC-δ | | Apoptosis | CASP-3 | Nitric oxide(Choudhari et al. 2013) |  |
|  |  | - |  |  |  | HIF-signaling pathway;  Erb-2 signaling pathway | | Angiogenesis  HIF-1α | VEGF – MMPs-IL-8 | Nitric oxide(Choudhari et al. 2013) |  |
|  |  | - |  |  |  |  |  | Inflammation | CXCL 10, CXCL11 | Paraquat(Paolillo et al. 2011) | Paraquat |
|  | 2 | | | | NADH dependent signaling pathway | Deregulated metabolism  Senescence | | MAP kinase, apoptosis | SIRT family(Huang et al. 2015) hTERT(Rathmell et al. 2015) | Ethanol(Dicker and Cederbaum 1992) | Ethanol |
| 2/3 | Morphological transformation | + Dysplasia  Anchorage-independent growth (carcinoma in situ) | Markers of cytoskeleton remodeling | Cell transformation assays available  SHE CTA (Vasseur and Lasne 2012) available in OECD(OECD 2015) (2015). However mechanistic understanding is currently considered insufficient by the OECD (OECD, 2015). | Actin polymerization and stabilization  Microtubules  Cell-cell and cell matrix adhesion  Organelle transport  Transcription and signal transduction | Gap junctional intercellular communication(Cruciani et al. 1997; Cruciani et al. 1999) c-myc(Maire et al. 2007)  Ornithine decarboxylase induction(Dhalluin et al. 1998; Dhalluin et al. 1997) | | Cell transformation  Connexins  Cadherins  β-catenin  Coronines  Cell adhesion | c-myc  cdh3  Ctnnbip1  Calml3  Coro1(Landkocz et al. 2011) | DEHP (Cruciani et al. 1999; Landkocz et al. 2011)  2,4-D(Maire et al. 2007) Chlordane(Bessi et al. 1995) | DEHP(Cruciani et al. 1999; Landkocz et al. 2011)  2,4-D(Maire et al. 2007)  Chlordane(Bessi et al. 1995) |
| 2/3 | Immunosuppression | - | Markers of chemokine signaling pathway | No available assay for regulatory purposes, but could the local lymph node assay (LLNA-TG 429- level 3/4) be modified, e.g. using the nude mouse model (which would represent a 3R’s refinement) as used for research purposes? | TGF-β signaling pathway | Transcription factor disruption examples include disruption of chemokine signaling, TGF-β, FAK, HIF-1a, IL-1a pathways, EGR, IGF-1, IL6(Goodson et al. 2015; Kravchenko et al. 2015) NF-kB | | Immunosuppression  Angiogenesis | IL-6 (Kravchenko et al. 2015) | Diethylhexyl  Phthalate(Ma et al. 2012) |  |
| 2 | Gene expression and cell signaling pathways | - | These modifications may result in either increased cell proliferation or selective cell death (apoptosis or necrosis). The release of calcium may result in the activation of kinases, such as PKC | Assays available  Can also be combined with epigenetic DNA methylation tests | Kinase; a cAMP-mediated cascade (Serezani et al., 2008)  Transcription factors, e.g., NFκB and AP-1. NFκB appears to be involved in the mediation of cell proliferation and apoptosis.  Changes in DNA methylation and hydroxymethylation patterns. | Calcium-calmodulin activated pathway  P\13K-Akt  Intracellular signal transducers such as nitric oxide | | Cytoskeleton restructuring (tumor microenvironment)  Apoptosis, necrosis | PKC  NFκB  AP-1 | Nitric oxide |  |
| 1/2 | Increased resistance to apoptotic cell death | - | Regulation of apoptosis mainly by BCL-2 family of mitochondria, death receptors and caspase network.  Intrinsic pathway: mitochondria mediated apoptosis (initiated by, e.g., high cytoplasmic Ca^+^ levels, ROS, xenobiotics) (Suh et al. 2013)  Extrinsic pathway: all non mitochondrial pathways | Assays available, e.g.,(Rieger et al. 2011) | ERα, P53, ErbB-/HER-2 tyrosine kinase, ERK, MAPK P16/P53, BCL-2/P53, PPARα, gap junction intracellular communication, hypersecretion of LH  Also ALK, VEGF, HER receptors, BRAF, Rho- associated protein kinase, fibroblast growth factor-9, cathepsins, cyclooxygenases, prostaglandins(Narayanan et al. 2015) | A KE with many downstream pathways.  Cross reaction with almost all the pathways indicated in this table. | |  | Annexin V (Rieger et al. 2011) | Lindane, BPA,  DBP and DEHP | Lindane |
| 2/3 | Pathogenic angiogenesis and neoangiogenesis  A multistep process | + or – model dependent, i.e :  Transition dependent | \| Endothelial cell activation in response to angiogenic factors \| \| --- \| | HTP assays available for primary angiogenic pathway molecular targets: VCAM, CXCL9 and HBD, CCL2, ICAM, uPAR, COLIII, CXL10, MMP1, AhR (role in cell cycle regulation)  miRNA target genes involved in angiogenesis(Caporali and Emanueli 2011)  Potential adaptation of Hen’s egg test-chorioallantoic membrane (HET-CAM) | Two key signaling pathways that interact: VEGFR- and TGF- mediated signaling pathways leading to tumor microenvironment and angiogenesis | Calcium signaling pathway – MAPK signaling pathway | Proliferation | Ras, Raf-1, ERK | Methylen-bisthiocianate (ICAM1, VCAM1) (Hu et al. 2015) | |  |
|  |  |  |  |  |  | Focal adhesion | Migration | FAK | Maneb | |  |
|  |  |  |  |  |  | P13k-AKt | Cell survival | CASP-9 – Bad | PFOS (VCAM1), Diniconazole (VCAM1) | |  |
|  |  |  |  |  |  | bFGF,  MMPs and urokinase, PDGF | Cell proliferation, migration and invasion. | MMP1 and MMP2, integrins, angiopoietin (Ang 1) |  | |  |
| 2 | Epigenetic mechanisms and associated genetic instability:  DNMT1  Histone modifications such as HATs and HDACs  Alteration of chromatin-modifying/remodeling activities  miRNA | - | DNA repair pathways (combination with gene expression)  Deregulation of *de novo* DNA methylation | Assays available for, e.g., DNA methylation, histone modifications(Greally and Jacobs 2013) | Caspase pathway, e.g., in colon carcinoma DCC(Forcet et al. 2001; Grady 2004; Mehlen and Fearon 2004) | Multiple cross interactions and mutual cross talk, e.g., inflammation, and inactivation of p53, to cell proliferation to invasion and metastasis | TGFβ signaling pathway | CASP-9 | Ethanol, arsenic(Davis et al. 2000)  DES (Newbold et al. 2006) | | Ethanol, arsenic, DES |
| 2 | Cellular senescence/ telomerase | - | Main mechanism is alteration of telomerase reverse transcriptase (hTERT).  Related to genetic instability (telomere-mediated chromosomal instability) | Expression of hTERT can be measured, but no specific assay available | hTERT pathway | ERG mediated signal, IL-6 | Senescence | hTERT protein expression  p21 | Phenobarbital(Martens et al. 1996) | | Phenobarbital(Martens et al. 1996) |
| 2/3 | Metastasis (migration, intra- and extravasation, survival outside of original tissue) | +  Anchorage independent growth/extravasion  Invasion/onset of metastasis | The ‘only hallmark definitively related to cancer’ (Hanahan and Weinberg 2011)  Ability of cells to grow in semisolid medium  Ability of cells to cross membrane barriers | Assays available(Albini and Noonan 2010) | Leukocyte transendothelial pathway | p53 signaling, MAP kinases,  VEGF signaling | Migration | CAMs | DES(Gibson and Saunders 2014)  TCDD(Murray et al. 2014) | | DES(Gibson and Saunders 2014)  TCDD(Murray et al. 2014) |

AhR, Aryl-hydrocarbon receptor; ARNT, AhR nuclear translocator; AP-1, activator protein 1; AR, androgen receptor; BCL-2, B-cell lymphoma 2 (oncogene); bFGF, basic fibroblast growth factor; BPA, bisphenol A; Calml3, calmodulin 3; CAMs, cell adhesion molecules; CASP, Caspase; cdh3, cadherin3; c-myc, myelocytomatosis oncogene; cttnbip1, catenin beta interacting protein1; CXCL, chemokine (C–X–C motif) ligand; DCC, deleted in colon carcinoma; DEHP, di(2-ethylhexyl)phthalate; DES, Diethylstilbestrol; DNMT1, DNA maintenance methyltransferase; ER, Estrogen Receptor; ERK, Extra signal-regulated kinases; FXR, Farnesoid x Receptor; GR, glucocorticoid receptor; HATs, histone acetyl transferases, HDACs, histone deacetylases; HIF-1α, hypoxia-inducible factor 1; Hsp, heat shock proteins, hTERT, human telomerase reverse transcriptase; IL, interleukin; ICAM1, intercellular adhesion molecule 1; JNK, c-Jun N-terminal kinases; LH, luteinizing hormone; MAPK, mitogen-activated protein kinase; MMP, matrix metalloproteinase; NF-κB, nuclear factor-κB; PAH, Polyaromatic hydrocarbon; PCBs, Polychlorinated biphenyls; PDGF, Platelet derived growth factor; PFOS, perfluorooctane sulfonate; PKC, protein kinase C; PPAR, peroxisome proliferator-activated receptor; RAR, retinoic acid receptor; ROS, reactive oxygen species; RXR, retinoid X receptor; SIRT, sirtuin; TCDD, 2,3,7,8-tetrachlorodibenzo-p-dioxin; THBD, thrombomodulin; TGF-β, transforming growth factor-β; TPA, 12-O-tetradecanoylphorbol-13-acetate; uPAR, urokinase-type plasminogen activator receptor; VCAM1, vascular cell-adhesion molecule 1; VDR, Vitamin D receptor; VEGF/VEGFR, vascular endothelial growth factor/receptor.

Updated from: Jacobs, M. N. *et al.* International regulatory needs for development of an IATA for non-genotoxic carcinogenic chemical substances. *Altex* **33**, 359-392, doi:10.14573/altex.1601201 (2016).

Albini A, Noonan DM (2010) The 'chemoinvasion' assay, 25 years and still going strong: the use of reconstituted basement membranes to study cell invasion and angiogenesis. Current opinion in cell biology 22(5):677-89 doi:10.1016/j.ceb.2010.08.017

Bessi H, Rast C, Rether B, Nguyen-Ba G, Vasseur P (1995) Synergistic effects of chlordane and TPA in multistage morphological transformation of SHE cells. Carcinogenesis 16(2):237-44 doi:10.1093/carcin/16.2.237

Biswas G, Srinivasan S, Anandatheerthavarada HK, Avadhani NG (2008) Dioxin-mediated tumor progression through activation of mitochondria-to-nucleus stress signaling. Proceedings of the National Academy of Sciences of the United States of America 105(1):186-91 doi:10.1073/pnas.0706183104

Black AT, Gray JP, Shakarjian MP, Laskin DL, Heck DE, Laskin JD (2008) Increased oxidative stress and antioxidant expression in mouse keratinocytes following exposure to paraquat. Toxicology and applied pharmacology 231(3):384-92 doi:10.1016/j.taap.2008.05.014

Caporali A, Emanueli C (2011) MicroRNA regulation in angiogenesis. Vascular pharmacology 55(4):79-86 doi:10.1016/j.vph.2011.06.006

Chang X, Lu W, Dou T, et al. (2013) Paraquat inhibits cell viability via enhanced oxidative stress and apoptosis in human neural progenitor cells. Chemico-biological interactions 206(2):248-55 doi:10.1016/j.cbi.2013.09.010

Choudhari SK, Chaudhary M, Bagde S, Gadbail AR, Joshi V (2013) Nitric oxide and cancer: a review. World journal of surgical oncology 11:118 doi:10.1186/1477-7819-11-118

Corton JC, Cunningham ML, Hummer BT, et al. (2014) Mode of action framework analysis for receptor-mediated toxicity: The peroxisome proliferator-activated receptor alpha (PPARalpha) as a case study. Critical reviews in toxicology 44(1):1-49 doi:10.3109/10408444.2013.835784

Cox MB, Miller CA, 3rd (2004) Cooperation of heat shock protein 90 and p23 in aryl hydrocarbon receptor signaling. Cell stress & chaperones 9(1):4-20 doi:10.1379/460.1

Cruciani V, Mikalsen SO, Vasseur P, Sanner T (1997) Effects of peroxisome proliferators and 12-O-tetradecanoyl phorbol-13-acetate on intercellular communication and connexin43 in two hamster fibroblast systems. International journal of cancer 73(2):240-8 doi:10.1002/(sici)1097-0215(19971009)73:2<240::aid-ijc14>3.0.co;2-j

Cruciani V, Rast C, Alexandre S, Nguyen-Ba G, Vasseur P (1999) Peroxisome Proliferator-induced Transformation of Syrian Hamster Embryo Cells: Influence of Experimental Procedures. Toxicology in vitro : an international journal published in association with BIBRA 13(3):445-57 doi:10.1016/s0887-2333(99)00016-8

Davis CD, Uthus EO, Finley JW (2000) Dietary selenium and arsenic affect DNA methylation in vitro in Caco-2 cells and in vivo in rat liver and colon. The Journal of nutrition 130(12):2903-9 doi:10.1093/jn/130.12.2903

DeBerardinis RJ, Chandel NS (2016) Fundamentals of cancer metabolism. Science advances 2(5):e1600200 doi:10.1126/sciadv.1600200

Dhalluin S, Elias Z, Cruciani V, et al. (1998) Two-stage exposure of Syrian-hamster-embryo cells to environmental carcinogens: superinduction of ornithine decarboxylase correlates with increase of morphological-transformation frequency. International journal of cancer 75(5):744-9 doi:10.1002/(sici)1097-0215(19980302)75:5<744::aid-ijc13>3.0.co;2-6

Dhalluin S, Gate L, Vasseur P, Tapiero H, Nguyen-Ba G (1997) Dysregulation of ornithine decarboxylase activity, apoptosis and Bcl-2 oncoprotein in Syrian hamster embryo cells stage-exposed to di(2-ethylhexyl)phthalate and tetradecanoylphorbol acetate. Carcinogenesis 18(11):2217-23 doi:10.1093/carcin/18.11.2217

Dicker E, Cederbaum AI (1992) Increased NADH-dependent production of reactive oxygen intermediates by microsomes after chronic ethanol consumption: comparisons with NADPH. Archives of biochemistry and biophysics 293(2):274-80 doi:10.1016/0003-9861(92)90395-d

Ekstrom G, Ingelman-Sundberg M (1989) Rat liver microsomal NADPH-supported oxidase activity and lipid peroxidation dependent on ethanol-inducible cytochrome P-450 (P-450IIE1). Biochemical pharmacology 38(8):1313-9 doi:10.1016/0006-2952(89)90338-9

Ferecatu I, Borot MC, Bossard C, et al. (2010) Polycyclic aromatic hydrocarbon components contribute to the mitochondria-antiapoptotic effect of fine particulate matter on human bronchial epithelial cells via the aryl hydrocarbon receptor. Particle and fibre toxicology 7:18 doi:10.1186/1743-8977-7-18

Forcet C, Ye X, Granger L, et al. (2001) The dependence receptor DCC (deleted in colorectal cancer) defines an alternative mechanism for caspase activation. Proceedings of the National Academy of Sciences of the United States of America 98(6):3416-21 doi:10.1073/pnas.051378298

Gibson DA, Saunders PT (2014) Endocrine disruption of oestrogen action and female reproductive tract cancers. Endocrine-related cancer 21(2):T13-31 doi:10.1530/erc-13-0342

Go RE, Hwang KA, Choi KC (2015) Cytochrome P450 1 family and cancers. The Journal of steroid biochemistry and molecular biology 147:24-30 doi:10.1016/j.jsbmb.2014.11.003

Goodson WH, 3rd, Lowe L, Carpenter DO, et al. (2015) Assessing the carcinogenic potential of low-dose exposures to chemical mixtures in the environment: the challenge ahead. Carcinogenesis 36 Suppl 1:S254-96 doi:10.1093/carcin/bgv039

Grady WM (2004) Genomic instability and colon cancer. Cancer metastasis reviews 23(1-2):11-27 doi:10.1023/a:1025861527711

Greally JM, Jacobs MN (2013) In vitro and in vivo testing methods of epigenomic endpoints for evaluating endocrine disruptors. Altex 30(4):445-71 doi:10.14573/altex.2013.4.445

Hanahan D, Weinberg RA (2011) Hallmarks of cancer: the next generation. Cell 144(5):646-74 doi:10.1016/j.cell.2011.02.013

Hu Z, Brooks SA, Dormoy V, et al. (2015) Assessing the carcinogenic potential of low-dose exposures to chemical mixtures in the environment: focus on the cancer hallmark of tumor angiogenesis. Carcinogenesis 36 Suppl 1:S184-202 doi:10.1093/carcin/bgv036

Huang G, Cui F, Yu F, et al. (2015) Sirtuin-4 (SIRT4) is downregulated and associated with some clinicopathological features in gastric adenocarcinoma. Biomedicine & pharmacotherapy = Biomedecine & pharmacotherapie 72:135-9 doi:10.1016/j.biopha.2015.04.013

Kravchenko J, Corsini E, Williams MA, et al. (2015) Chemical compounds from anthropogenic environment and immune evasion mechanisms: potential interactions. Carcinogenesis 36 Suppl 1:S111-27 doi:10.1093/carcin/bgv033

Kudryavtseva AV, Krasnov GS, Dmitriev AA, et al. (2016) Mitochondrial dysfunction and oxidative stress in aging and cancer. Oncotarget 7(29):44879-44905 doi:10.18632/oncotarget.9821

Kukielka E, Cederbaum AI (1992) The effect of chronic ethanol consumption on NADH- and NADPH-dependent generation of reactive oxygen intermediates by isolated rat liver nuclei. Alcohol and alcoholism (Oxford, Oxfordshire) 27(3):233-9

Landkocz Y, Poupin P, Atienzar F, Vasseur P (2011) Transcriptomic effects of di-(2-ethylhexyl)-phthalate in Syrian hamster embryo cells: an important role of early cytoskeleton disturbances in carcinogenesis? BMC genomics 12:524 doi:10.1186/1471-2164-12-524

Larigot L, Juricek L, Dairou J, Coumoul X (2018) AhR signaling pathways and regulatory functions. Biochimie open 7:1-9 doi:10.1016/j.biopen.2018.05.001

Lin LC, Hsu SL, Wu CL, Hsueh CM (2014) TGFbeta can stimulate the p(38)/beta-catenin/PPARgamma signaling pathway to promote the EMT, invasion and migration of non-small cell lung cancer (H460 cells). Clinical & experimental metastasis 31(8):881-95 doi:10.1007/s10585-014-9677-y

Lucas IK, Kolodziej H (2015) Trans-Resveratrol Induces Apoptosis through ROS-Triggered Mitochondria-Dependent Pathways in A549 Human Lung Adenocarcinoma Epithelial Cells. Planta medica 81(12-13):1038-44 doi:10.1055/s-0035-1546129

Ma H, Li J, Yang JJ, et al. (2012) [Di-2-ethylhexyl phthalate and its metabolite single-ethylhexyl phthalate affect TGF-beta 1 expression and telomerase activity in the testis of young male rats]. Zhonghua nan ke xue = National journal of andrology 18(9):783-8

Maire MA, Rast C, Landkocz Y, Vasseur P (2007) 2,4-Dichlorophenoxyacetic acid: effects on Syrian hamster embryo (SHE) cell transformation, c-Myc expression, DNA damage and apoptosis. Mutation research 631(2):124-36 doi:10.1016/j.mrgentox.2007.03.008

Martens U, Lennartsson P, Hogberg J, Stenius U (1996) Low expression of the WAF1/CIP1 gene product, p21, in enzyme-altered foci induced in rat liver by diethylnitrosamine or phenobarbital. Cancer letters 104(1):21-6 doi:10.1016/0304-3835(96)04218-8

Mehlen P, Fearon ER (2004) Role of the dependence receptor DCC in colorectal cancer pathogenesis. Journal of clinical oncology : official journal of the American Society of Clinical Oncology 22(16):3420-8 doi:10.1200/jco.2004.02.019

Murphy KA, Villano CM, Dorn R, White LA (2004) Interaction between the aryl hydrocarbon receptor and retinoic acid pathways increases matrix metalloproteinase-1 expression in keratinocytes. The Journal of biological chemistry 279(24):25284-93 doi:10.1074/jbc.M402168200

Murray IA, Patterson AD, Perdew GH (2014) Aryl hydrocarbon receptor ligands in cancer: friend and foe. Nature reviews Cancer 14(12):801-14 doi:10.1038/nrc3846

Nakanishi K, Sakamoto M, Yamasaki S, Todo S, Hirohashi S (2005) Akt phosphorylation is a risk factor for early disease recurrence and poor prognosis in hepatocellular carcinoma. Cancer 103(2):307-12 doi:10.1002/cncr.20774

Narayanan KB, Ali M, Barclay BJ, et al. (2015) Disruptive environmental chemicals and cellular mechanisms that confer resistance to cell death. Carcinogenesis 36 Suppl 1:S89-110 doi:10.1093/carcin/bgv032

Newbold RR, Padilla-Banks E, Jefferson WN (2006) Adverse effects of the model environmental estrogen diethylstilbestrol are transmitted to subsequent generations. Endocrinology 147(6 Suppl):S11-7 doi:10.1210/en.2005-1164

Nieminen T, Toivanen PI, Rintanen N, et al. (2014) The impact of the receptor binding profiles of the vascular endothelial growth factors on their angiogenic features. Biochimica et biophysica acta 1840(1):454-63 doi:10.1016/j.bbagen.2013.10.005

OECD (2015) Guidance Document on the in vitro syrian hamster embryo (SHE) Cell Transformation Assay. . Series on Testing and Assessment 214. . OECD, Paris

Paolillo N, Piccirilli S, Giardina E, Rispoli V, Colica C, Nistico S (2011) Effects of paraquat and capsaicin on the expression of genes related to inflammatory, immune responses and cell death in immortalized human HaCat keratinocytes. International journal of immunopathology and pharmacology 24(4):861-8 doi:10.1177/039463201102400405

Parke DV (1994) The cytochromes P450 and mechanisms of chemical carcinogenesis. Environmental health perspectives 102(10):852-3 doi:10.1289/ehp.94102852

Ralph SJ, Rodriguez-Enriquez S, Neuzil J, Saavedra E, Moreno-Sanchez R (2010) The causes of cancer revisited: "mitochondrial malignancy" and ROS-induced oncogenic transformation - why mitochondria are targets for cancer therapy. Molecular aspects of medicine 31(2):145-70 doi:10.1016/j.mam.2010.02.008

Rathmell KW, Chen F, Creighton CJ (2015) Genomics of chromophobe renal cell carcinoma: implications from a rare tumor for pan-cancer studies. Oncoscience 2(2):81-90 doi:10.18632/oncoscience.130

Rieger AM, Nelson KL, Konowalchuk JD, Barreda DR (2011) Modified annexin V/propidium iodide apoptosis assay for accurate assessment of cell death. Journal of visualized experiments : JoVE(50) doi:10.3791/2597

Schaap MM, Wackers PF, Zwart EP, et al. (2015) A novel toxicogenomics-based approach to categorize (non-)genotoxic carcinogens. Archives of toxicology 89(12):2413-27 doi:10.1007/s00204-014-1368-6

Senft D, Ronai ZA (2016) Regulators of mitochondrial dynamics in cancer. Current opinion in cell biology 39:43-52 doi:10.1016/j.ceb.2016.02.001

Sever R, Glass CK (2013) Signaling by nuclear receptors. Cold Spring Harbor perspectives in biology 5(3):a016709 doi:10.1101/cshperspect.a016709

Skeberdis VA, Lan J, Opitz T, Zheng X, Bennett MV, Zukin RS (2001) mGluR1-mediated potentiation of NMDA receptors involves a rise in intracellular calcium and activation of protein kinase C. Neuropharmacology 40(7):856-65 doi:10.1016/s0028-3908(01)00005-3

Song H, Zhang T, Yang P, et al. (2015) Low doses of bisphenol A stimulate the proliferation of breast cancer cells via ERK1/2/ERRgamma signals. Toxicology in vitro : an international journal published in association with BIBRA 30(1 Pt B):521-8 doi:10.1016/j.tiv.2015.09.009

Suh DH, Kim MK, Kim HS, Chung HH, Song YS (2013) Mitochondrial permeability transition pore as a selective target for anti-cancer therapy. Frontiers in oncology 3:41 doi:10.3389/fonc.2013.00041

Swierenga SH, Yamasaki H (1992) Performance of tests for cell transformation and gap-junction intercellular communication for detecting nongenotoxic carcinogenic activity. IARC scientific publications(116):165-93

Vasseur P, Lasne C (2012) OECD Detailed Review Paper (DRP) number 31 on "Cell Transformation Assays for Detection of Chemical Carcinogens": main results and conclusions. Mutation research 744(1):8-11 doi:10.1016/j.mrgentox.2011.11.007

Wong RL, Walker CL (2013) Molecular pathways: environmental estrogens activate nongenomic signaling to developmentally reprogram the epigenome. Clinical cancer research : an official journal of the American Association for Cancer Research 19(14):3732-7 doi:10.1158/1078-0432.Ccr-13-0021

Yang W, Tiffany-Castiglioni E (2008) Paraquat-induced apoptosis in human neuroblastoma SH-SY5Y cells: involvement of p53 and mitochondria. Journal of toxicology and environmental health Part A 71(4):289-99 doi:10.1080/15287390701738467
